# Does Lower Regression Error Mean Stronger Forensic Evidence? Machine Learning Regression Versus Demirjian and Willems Methods for Dental Age Estimation at 12- and 15-Year Legal Thresholds

**DOI:** 10.3390/diagnostics16172690

**Published:** 2026-08-23

**Authors:** Mustafa Doğan, Muhammed Emin Parlak, Kadir Sezer Koçak, Yasin Etli, Bora Özdemir, Katibe Tuğçe Temur

**Affiliations:** 1 Department of Forensic Medicine, Faculty of Medicine, Niğde Ömer Halisdemir University, 51000 Nigde, Türkiye; 2 Niğde Forensic Medicine Branch Office, The Council of Forensic Medicine, Ministry of Justice, 51000 Nigde, Türkiye; 3 Department of Forensic Medicine, Faculty of Medicine, Van Yuzuncu Yil University, 65000 Van, Türkiye; 4 Department of Oral and Maxillofacial Radiology, Faculty of Dentistry, Niğde Ömer Halisdemir University, 51000 Nigde, Türkiye

**Keywords:** forensic dentistry, dental age estimation, machine learning, Demirjian method, Willems method, legal age thresholds, likelihood ratio, criminal responsibility

## Abstract

**Background/Objectives**: Dental age estimation is important in clinical and forensic practice, particularly when skeletal indicators are unavailable or compromised. Machine-learning models often achieve lower regression errors than conventional dental methods; however, whether this translates into better classification performance at legally relevant age thresholds remains unclear. This study compared the Demirjian and Willems methods with several machine learning models for overall accuracy and threshold-specific performance at the jurisdiction-specific ages of 12 and 15 years. **Methods**: A total of 1384 panoramic radiographs from individuals aged 8.00–15.99 years were retrospectively evaluated. The developmental stages of the seven left mandibular permanent teeth and sex were used as model inputs. Linear Regression, Decision Tree, Random Forest, Support Vector Regression, Multilayer Perceptron, Gradient Boosting, and XGBoost were trained using cross-validation and evaluated on an internal holdout set. Performance was assessed using regression errors, age-group-specific bias, sensitivity, specificity, balanced accuracy, and likelihood ratios. **Results**: Machine-learning models generally produced lower errors than conventional methods. In the holdout set, the lowest mean absolute error was 0.512 years for Support Vector Regression and Gradient Boosting, followed by 0.519 years for XGBoost, compared with 0.649 and 0.654 years for the Willems and Demirjian methods. However, lower regression error did not consistently improve threshold-specific performance. At 12 years, machine learning models increased sensitivity but reduced specificity and positive likelihood ratios relative to Willems. At 15 years, Linear Regression and Random Forest produced no positive predictions, whereas the better-performing models showed results similar to Willems. **Conclusions**: Lower regression error does not necessarily indicate better forensic threshold-specific classification performance. Dental age-estimation models should therefore be validated using threshold-specific likelihood ratios, classification metrics, and age-group-specific bias in addition to overall prediction errors.

## 1. Introduction

Estimated age is an important component of an individual’s biological identity and should be distinguished from chronological age. In living individuals, chronological age generally corresponds to calendar age, whereas biological or physiological age reflects both the stage of growth associated with the maturation of different tissues and organs and the individual’s ageing process [1]. In this context, dental age estimation is widely used to estimate an individual’s age in archaeology, anthropology, medicine, and both clinical and forensic dentistry [2]. In forensic settings, it is particularly important for individuals who lack valid identity documents and may be required to resolve criminal, civil, or administrative issues related to age determination, asylum procedures, retirement eligibility, or the identification of victims of mass disasters [3,4].

In living individuals, dental age estimation based on dental development can be applied from childhood through young adulthood, until the maturation of all teeth is complete. This period represents the upper age limit that can be assessed on the basis of dental maturation. Therefore, age-estimation methods should be as reliable as possible [5,6]. Dental age-estimation techniques are widely used because dental development is considerably less affected by environmental factors, such as premature loss of deciduous teeth, dental crowding, or caries, than skeletal development [7]. They are also preferred because dental structures can be readily evaluated using radiographic imaging.

Various methods have been developed for dental age estimation, most of which are based on radiographic or tomographic imaging. Because many of these techniques assess the developmental stages of the permanent teeth, their use is largely limited to children and adolescents. One of the most widely used approaches is the Demirjian method, which classifies the seven left mandibular permanent teeth into eight developmental stages, from A to H. In this method, the mineralization stage and developmental status of each tooth are assigned scores according to a statistical model [8]. The Willems method is fundamentally based on the Demirjian approach; however, its scoring tables were modified for the Belgian population, and the resulting estimates are expressed directly in years [9].

Advances in artificial intelligence (AI), particularly in machine learning (ML) algorithms, have created new opportunities to automate and improve dental age estimation. By analysing large, multidimensional datasets, ML techniques can identify patterns that may be difficult to detect using conventional statistical approaches or human observation [10]. Machine learning encompasses a broad family of algorithms with varying levels of complexity, ranging from readily interpretable models, such as linear and polynomial regression, to more complex approaches, including support vector machines and neural networks. Models may be developed using supervised learning, in which the training data are labelled, or unsupervised learning, in which the model independently extracts information and patterns from unlabelled data [11].

In this context, Abuabara et al. (2025) [12] applied machine learning methods to manually assigned Demirjian developmental stage scores for dental age estimation. Among individuals aged 7–16 years, the mean absolute error (MAE), which was 1.34 years with the conventional assessment, was reduced to below 1 year using ML methods, yielding improved estimates without relying on conventional conversion tables [12]. Galibourg et al. (2021) [13] evaluated a modified Demirjian staging system using ML methods by incorporating the seven left mandibular teeth and all four third molars, and reported MAE values of 0.729 years for individuals aged 4–16 years and 1.166 years for those aged 4–24 years [13]. Guo et al. (2024) [14] further adapted the Demirjian method by including third molars in the model, thereby extending its applicability from the original age range of 3–16 years to 5.00–24.99 years [14]. These approaches may facilitate the development of population-specific tools and enable more accurate calibration of reference standards for the target population [4,15]. Collectively, these findings demonstrate the potential of ML-enhanced models and underscore the need for further validation across diverse populations.

The meta-analysis by Niño-Sandoval et al. (2024) [4] highlighted differences in error rates between manual approaches and ML techniques. The reviewed studies generally showed that ML methods achieved greater accuracy than conventional radiological approaches, while also emphasizing the need for more comprehensive comparative studies to better define their capabilities and limitations [4]. Nevertheless, an important methodological limitation of regression-based age estimation is its tendency to overestimate age in younger individuals and underestimate age in older individuals when applied across different age groups. This may lead to a minor being incorrectly classified as an adult, or an adult as a minor, with potentially adverse legal consequences [16]. Regression methods may also produce a bias known as age mimicry or regression toward the mean, whereby estimated ages are drawn toward the mean age of the reference sample rather than accurately reflecting chronological age [17]. Lee and Chen (2025) [18] described this phenomenon in ML regression models as the “systematic bias of machine learning” [18]. Furthermore, when the age distribution of the target sample differs from that of the reference sample, age estimates may become biased toward the age distribution of the reference population [17].

Beyond statistical accuracy, forensic use of age-estimation models raises the question of what constitutes an ethically and technically acceptable error at a legally relevant threshold. International forensic guidance generally avoids a fixed numerical tolerance and instead adopts an asymmetric decision framework: the minimum-age concept derives a conservative lower-bound estimate so that a threshold is considered surpassed only with probability bordering on certainty, explicitly to avoid misclassifying a minor as an adult [19,20]. A comparable “benefit of the doubt” principle applies in international child-protection and asylum practice [21]. This asymmetry—tolerating false negatives more readily than false positives at a legal threshold—provides the interpretive frame we return to when discussing threshold-specific sensitivity and specificity below.

Despite the growing global interest in ML applications for dental age estimation, most existing studies evaluate model performance using overall regression metrics, such as MAE, RMSE, and R^2^. By learning the relationship between dental developmental stages and chronological age directly from the target population, stage-based ML models effectively provide a population-specific remapping of the conventional stage-to-age relationship. These models do not learn features directly from the radiographic images; therefore, their performance remains constrained by the age-related information contained in the manually assigned developmental stages. However, lower regression error after such recalibration does not necessarily mean that individuals are more accurately placed on the appropriate side of a legally relevant age threshold. At these thresholds, false-positive and false-negative classifications may have different consequences; therefore, model performance should be assessed not only in terms of overall predictive accuracy but also using sensitivity, specificity, balanced accuracy, and diagnostic likelihood-ratio measures. Nevertheless, studies that jointly compare conventional dental age-estimation methods and ML-based, population-specific models in terms of both overall regression performance and their ability to correctly distinguish individuals below versus at or above legal age thresholds remain limited. Accordingly, this study aimed to compare the Demirjian and Willems methods with several ML regression models in terms of overall regression performance and threshold-specific performance at the ages of 12 and 15 years, as defined in the Turkish Penal Code, and to determine whether the reduction in prediction error achieved through population-specific ML modelling translates into more accurate placement of individuals relative to these legally relevant age thresholds.

## 2. Materials and Methods

### 2.1. Ethical Approval

This study was approved by the Non-Interventional Clinical Research Ethics Committee of Niğde Ömer Halisdemir University, Türkiye, on 26 February 2026 (protocol no. 2026/36). The study was conducted in accordance with the principles of the Declaration of Helsinki.

### 2.2. Study Sample

Panoramic radiographs of patients who received care at the Oral and Dental Health Research and Application Center of Niğde Ömer Halisdemir University between January 2022 and January 2026 were retrospectively reviewed. All digital panoramic radiographs were acquired using the same device to ensure consistent, high-quality imaging. The images included in the study were obtained using a PLANMECA ProMax DIMAX 2 system (Planmeca Oy, Helsinki, Finland) with exposure parameters of 64 kV, 6.3 mA, and 15.8 s. To enhance the reliability of the study, only panoramic radiographs classified as Grade 1 according to the criteria of the United Kingdom National Radiological Protection Board were evaluated [22].

A total of 1384 children and adolescents aged 8.00–15.99 years were included in the study. Participants were stratified by age and sex. The final sample comprised 725 females and 659 males. Digital orthopantomograms (OPGs) were classified into eight age groups according to each individual’s chronological age.

**Figure 1:**
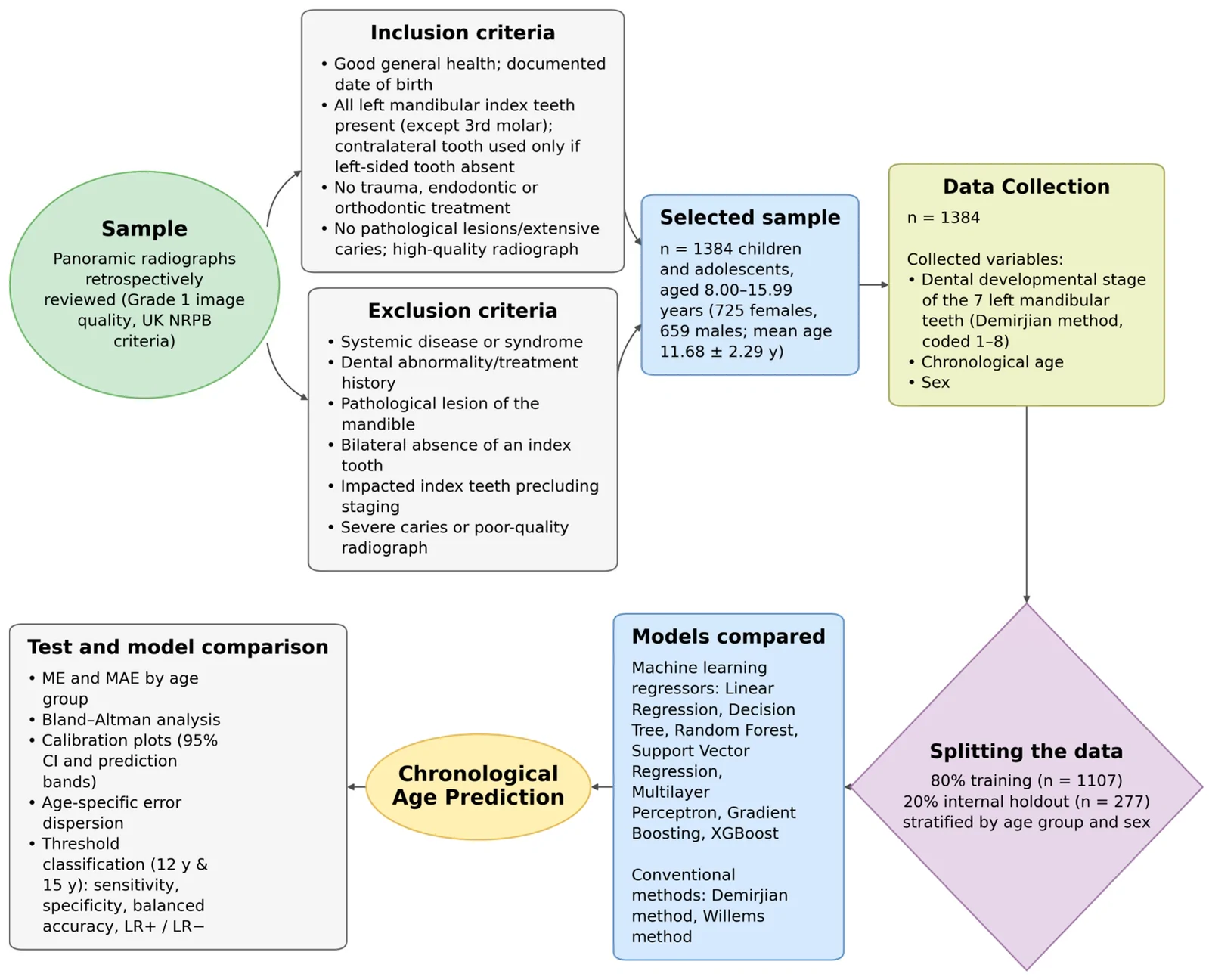
Overview of the study workflow.

Children were included if they were in good general health, had a documented date of birth, had all mandibular index teeth available for assessment except the third molar, and had no history of dental trauma or endodontic or orthodontic treatment. Individuals were also required to have no pathological lesions, such as tumors or cysts, no extensive caries, and high-quality radiographs. Children with systemic diseases or syndromes, dental abnormalities or a history of dental treatment, pathological lesions of the mandible, bilateral absence of an index tooth, impacted index teeth that precluded reliable developmental staging, severe caries, or poor-quality radiographs were excluded [23]. Owing to the retrospective design of the study, no additional imaging was performed for research purposes, and all data were anonymized before analysis. The overall study workflow—including the sample-selection criteria, the resulting analytic sample, the training/internal-holdout split, and the models and evaluation metrics compared—is summarized in Figure 1.

### 2.3. Observer Characteristics and Intra- and Interobserver Agreement

Dental developmental stages were assessed by two forensic medicine specialists (MD and KSK), who were trained by an oral and maxillofacial radiologist with 10 years of professional experience (KTT). Following the training period, MD and KSK jointly evaluated all radiographs, and the developmental stage scores used in the main dataset were assigned by consensus. Observer reliability was assessed using 60 randomly selected radiographs, corresponding to 420 tooth-stage observations. For intraobserver reliability, KSK independently evaluated the selected radiographs on two separate occasions one month apart. For interobserver reliability, the same radiographs were independently evaluated by KTT. Because the Demirjian A–H stages represent ordered developmental categories, agreement was assessed using linearly weighted Cohen’s kappa, following the approach described by Abdul Rahim et al. [24]. Kappa coefficients were calculated in Python 3.12.13 (Python Software Foundation, Wilmington, DE, USA) using scikit-learn 1.6.1 in the Google Colaboratory environment (Google LLC, Mountain View, CA, USA). Ninety-five percent confidence intervals were estimated using 20,000 bootstrap resamples performed at the radiograph level to account for the clustering of the seven tooth-stage observations within each radiograph.

### 2.4. Calculation of Chronological Age and Dental Age Estimation

Chronological age (CA) was calculated by dividing the number of days between the date of birth and the date of panoramic radiograph acquisition by 365.25 [12]. Age values were recorded in decimal years.

The seven left mandibular permanent teeth (central incisor to second molar) were assessed because they constitute the standard reference set defined in the original Demirjian method and retained in the Willems modification; the same tooth set was used as input to the machine learning models to preserve methodological comparability between the conventional and machine learning approaches.

The developmental stage of each of the seven left mandibular permanent teeth, from the central incisor to the second molar, was determined according to the eight radiographic stages described by Demirjian et al. [8]. Stages A–D represented crown maturation, whereas stages E–H represented root maturation. When a left mandibular index tooth was absent, the corresponding contralateral homologous tooth was evaluated, as described in previous stage-based dental age-estimation studies [12,13]. Contralateral teeth were used only as substitutes for absent left-sided index teeth; bilateral assessment was not routinely performed. The sum of the resulting scores constituted the individual dental maturity score, which was subsequently converted into dental age. Willems dental age was calculated using the modified scoring tables developed by Willems et al. [9].

For use as input variables in the ML models, the alphabetical Demirjian stages were converted into sequential numerical scores, adapting and simplifying the ordinal encoding approach used by Galibourg et al. [13]. Because only the conventional A–H Demirjian stages were included in the present study, they were coded consecutively from 1 to 8 (A = 1, B = 2, …, H = 8), thereby preserving their developmental order. Sex was additionally encoded as female = 0 and male = 1.

### 2.5. Machine Learning Algorithms

The developmental stages of the seven permanent teeth in the left mandibular region were converted into numerical variables and used as model inputs together with sex. Accordingly, the ML approach used in this study was stage-based rather than image-based. Using these predictors, several machine learning regression models with different algorithmic structures were trained and evaluated to estimate chronological age. The models included Linear Regression (LR), Decision Tree Regressor (DT), Random Forest Regressor (RF), Support Vector Regression (SVR), Multilayer Perceptron Regressor (MLP), Gradient Boosting Regressor (GB), and Extreme Gradient Boosting Regressor (XGB). These algorithms were selected to represent linear relationships, tree-based decision structures, kernel-based nonlinear patterns, ensemble learning, and artificial neural network-based associations.

Linear Regression (LR) is a fundamental regression approach that models the linear relationship between a dependent variable and one or more independent variables. It was included as a highly interpretable reference model. The Decision Tree (DT) algorithm generates predictions by recursively partitioning the dataset into increasingly homogeneous subgroups according to a series of decision rules. In regression tasks, the mean value of the observations within each terminal node is used as the predicted outcome [25]. Random Forest (RF) is an ensemble-based regression method that combines multiple decision trees. Numerous regression trees are trained on different subsets of the data generated through bootstrap sampling, and the final prediction is obtained by averaging the predictions produced by the individual trees. Compared with a single decision tree, this structure reduces variance, improves model stability, and limits the risk of overfitting [25]. Support Vector Regression (SVR) is a regression method that controls model complexity while attempting to keep prediction errors within a predefined tolerance margin. It is particularly useful when nonlinear relationships are present and the patterns among variables cannot be adequately represented by conventional linear models [26]. The Multilayer Perceptron (MLP) is a feedforward artificial neural network composed of an input layer, one or more hidden layers, and an output layer. The connection weights between layers are iteratively updated during training to minimize prediction error. This architecture enables the modelling of complex and nonlinear relationships among variables [27]. Gradient Boosting (GB) is an ensemble-learning approach in which weak learners are combined sequentially. Each new decision tree is trained to reduce the errors made by the preceding trees, allowing the model to focus on residual errors at each iteration and progressively improve predictive performance [28]. Extreme Gradient Boosting (XGB) is an ensemble-learning algorithm based on the gradient-boosting principle but enhanced in terms of computational efficiency, regularization, and parallel processing. By sequentially adding weak learners and incorporating regularization mechanisms, XGB aims to reduce overfitting while maintaining high predictive performance [29].

### 2.6. Training, Cross-Validation, and Testing Procedure

Hyperparameter optimization was performed to improve model performance and identify the optimal configuration for each algorithm. Grid search was used for this purpose. In this approach, predefined combinations of hyperparameters are systematically evaluated for each model, and the optimal parameter set is selected based on cross-validation performance [30].

The dataset was divided into training and internal holdout test subsets. Eighty percent of the data were allocated to the training set for model development and hyperparameter optimization, whereas the remaining 20% constituted an internal holdout test set for final performance evaluation. The holdout observations were not used during model development or hyperparameter tuning and therefore provided an internal assessment of predictive performance on previously unseen observations.

Stratified five-fold cross-validation was applied within the training set for model selection and hyperparameter optimization. Stratification was performed according to sex and age group to ensure that each fold represented the age and sex distribution of the original dataset as closely as possible. Model selection was based on mean squared error (MSE), using negative mean squared error as the GridSearchCV refit criterion. A random seed of 44 was used for data splitting and cross-validation. During cross-validation, the training data were divided into five approximately equal subsets. In each iteration, four subsets were used to train the model, while the remaining subset was used for validation. This procedure was repeated five times, with each subset serving once as the validation set. Consequently, model performance was evaluated across multiple training–validation scenarios rather than relying on a single data split, thereby enabling more robust model selection [31,32].

After hyperparameter optimization, each model was retrained on the entire training set using the best-performing parameter combination, and its final performance was evaluated on the internal holdout test set. Accordingly, cross-validation-based model development performance and internal holdout test performance were reported separately. Detailed information on preprocessing, feature scaling, hyperparameter search spaces, optimization criteria, random seeds, software versions, and the final selected settings for each model is provided in Supplementary Table S1.

### 2.7. Data Analysis and Model Evaluation Metrics

Model performance was evaluated in terms of both overall regression accuracy and threshold-specific forensic performance at legally relevant age thresholds. The Demirjian and Willems methods were included as conventional dental age-estimation approaches and were compared with the machine learning models using the same dataset.

Overall regression performance was assessed based on the differences between chronological age and predicted age. For this purpose, mean error (ME), mean absolute error (MAE), root mean squared error (RMSE), and the coefficient of determination (R^2^) were calculated. ME was defined as the difference between predicted age and chronological age and was used to assess systematic overestimation or underestimation. A positive ME indicated age overestimation, whereas a negative ME indicated age underestimation. MAE and RMSE were used to evaluate predictive accuracy, while R^2^ represented the proportion of variance in chronological age explained by the model predictions. In addition, scatter plots were generated to visually assess the relationship between actual and predicted ages and the deviations observed across methods.

To statistically compare the predictive performance of the Demirjian and Willems methods with that of the ML models, paired comparisons were performed at the individual level. For each participant, the absolute errors produced by the conventional method and the corresponding ML model were compared. Differences in MAE were evaluated using bootstrap resampling with 1000 repetitions. Ninety-five percent confidence intervals were calculated from the 2.5th and 97.5th percentiles of the bootstrap distribution. A confidence interval that did not include zero was interpreted as indicating a statistically significant difference in MAE between the two methods. These analyses were conducted separately for each ML model against both the Demirjian and Willems methods.

Age-group-specific error analyses were performed to examine age-related systematic error patterns in the models. Age groups were defined as one-year intervals according to chronological age. For each model and age group, mean error and mean absolute error were calculated together with their 95% confidence intervals using bootstrap resampling with 1000 repetitions. A mean-error confidence interval that did not include zero was interpreted as evidence of statistically significant systematic bias within the corresponding age group. A positive mean error indicated overestimation of chronological age, whereas a negative mean error indicated underestimation.

Threshold-specific forensic performance analyses were conducted at the ages of 12 and 15 years, taking into account the provisions of the Turkish Penal Code concerning criminal responsibility in children [33]. Article 31 of the Turkish Penal Code stipulates that children who have not completed 12 years of age at the time of the offence have no criminal responsibility. For children who have completed 12 years but not 15 years of age, the ability to understand the legal meaning and consequences of the act and to control their behaviour must be evaluated. Accordingly, the 12- and 15-year thresholds were selected for threshold-specific forensic performance analyses.

For the threshold analyses, individuals were classified as “≥12 years” or “<12 years” at the 12-year threshold and as “≥15 years” or “<15 years” at the 15-year threshold. At each threshold, chronological age was used to divide individuals into two groups: those at or above the threshold age and those below it. Likewise, the age predicted by each model was classified as positive or negative according to the corresponding threshold. The upper age limit was selected to remain within the developmental range primarily addressed by the conventional seven-tooth Demirjian–Willems framework and to maintain direct comparability with previous stage-based ML studies using the same mandibular tooth set [8,9,12,13]. Because the study sample was restricted to individuals aged 8.00–15.99 years, the ≥15-year category comprised only individuals aged 15.00–15.99 years and did not include individuals aged 16 years or older. Accordingly, the 15-year threshold analysis was considered exploratory and was interpreted as an assessment of classification performance near the upper boundary of the study age range. Based on these classifications, the numbers of true positives (TP), false negatives (FN), false positives (FP), and true negatives (TN) were calculated. Sensitivity, specificity, and balanced accuracy (BA) were then derived. Sensitivity represented the proportion of individuals at or above the threshold age who were correctly classified as positive, whereas specificity represented the proportion of individuals below the threshold age who were correctly classified as negative. Balanced accuracy was calculated as the mean of sensitivity and specificity.

Diagnostic likelihood-ratio analysis was performed as an additional measure of threshold-specific binary classification performance. For each age threshold, individuals were classified according to whether their chronological age was equal to or above the threshold or below the threshold. Likewise, model predictions were classified as positive (predicted age ≥ threshold) or negative (predicted age < threshold). The positive likelihood ratio (LR+) was calculated as the probability of a positive classification among individuals truly at or above the threshold divided by the probability of the same classification among individuals below the threshold. The negative likelihood ratio (LR−) was calculated analogously for negative classifications. For ease of interpretation of below-threshold classifications, the reciprocal of LR− (LRbelow = 1/LR−) was additionally reported. When the denominator was zero or the corresponding prediction category was not observed, the likelihood ratio was reported as “not estimable” (NE). The use of likelihood-ratio reasoning was conceptually inspired by the general framework of forensic evaluative reporting described by ENFSI [34]; however, the LR+ and LR− values calculated in the present study were used as population-level diagnostic classification measures and not as case-specific evaluative likelihood ratios in the forensic sense.

The metrics were calculated using the following formulas:

Sensitivity = TP/(TP + FN)

Specificity = TN/(TN + FP)

Balanced accuracy = (Sensitivity + Specificity)/2

LR+ = Sensitivity/(1 − Specificity)

LR− = (1 − Sensitivity)/Specificity

LRbelow = 1/LR−

Here, LR+ summarizes the discriminatory performance of an at-or-above-threshold classification, whereas LRbelow summarizes the discriminatory performance of a below-threshold classification. These measures describe classification performance at the population level rather than the evidential strength of an individual forensic case.

Threshold-specific performance comparisons were also conducted using a paired bootstrap approach at the individual level. The Demirjian and Willems methods were compared separately with each ML model at the 12- and 15-year thresholds. Bootstrap confidence intervals based on 1000 repetitions were calculated for differences in sensitivity, specificity, balanced accuracy, log10-transformed LR+, and log10-transformed LRbelow. Because likelihood ratios may exhibit right-skewed distributions, comparisons were performed after log10 transformation. A confidence interval that did not include zero was interpreted as indicating a statistically significant difference in the corresponding performance measure.

Because multiple paired comparisons were performed across models, thresholds, validation splits, and correlated performance measures, no formal multiplicity adjustment was applied. These analyses were considered exploratory and were intended primarily to characterize the magnitude, direction, and consistency of comparative performance rather than to support confirmatory hypothesis testing. Accordingly, 95% confidence intervals were reported to quantify uncertainty, and interpretation emphasized effect magnitude and consistency across models and validation splits rather than binary statistical significance alone.

To assess method agreement beyond pooled error metrics, Bland–Altman analysis was performed for each method by plotting the difference between predicted age and chronological age against their mean; bias and 95% limits of agreement (bias ± 1.96 × SD) were calculated separately for the out-of-fold and internal holdout predictions. Overall regression calibration was additionally examined by regressing predicted age on chronological age using ordinary least-squares regression, with 95% confidence bands for the fitted mean relationship and 95% prediction bands for individual observations. To characterize age-dependent prediction behavior, residuals were also evaluated separately within one-year chronological-age strata. For each stratum, the mean prediction error and its bootstrap 95% confidence interval were calculated, together with descriptive residual dispersion defined as the age-specific mean error ± 1.96 SD. These dispersion limits were treated as descriptive measures of within-stratum error variability rather than as validated prediction intervals for new individuals. Threshold-classification profiles were additionally calculated by chronological-age stratum for the 12- and 15-year legal thresholds, with exact (Clopper–Pearson) binomial 95% confidence intervals. Full methodological details and supplementary figures are provided in Supplementary File S2.

All analyses were conducted separately for out-of-fold predictions from the training set and predictions from the internal holdout test set. Out-of-fold predictions represented estimates generated for each individual by a model that had not been trained on that individual during cross-validation. Holdout predictions were obtained from observations that were withheld from model development and hyperparameter optimization. This approach enabled the separate assessment of cross-validation performance during model development and internal validation performance on previously unseen observations.

All machine learning, bootstrap, and statistical evaluation analyses were performed using the Python programming language (version 3.12.13). The machine learning models were trained in the Google Colaboratory environment using the scikit-learn (version 1.6.1) and XGBoost (version 3.3.0) libraries. NumPy (version 2.0.2), pandas (version 2.2.2), and SciPy (version 1.16.3) were used for numerical and statistical computation. Performance metrics, bootstrap confidence intervals, and threshold-specific forensic analyses were calculated using custom-developed Python code.

## 3. Results

### 3.1. Sample Characteristics

**Figure 2:**
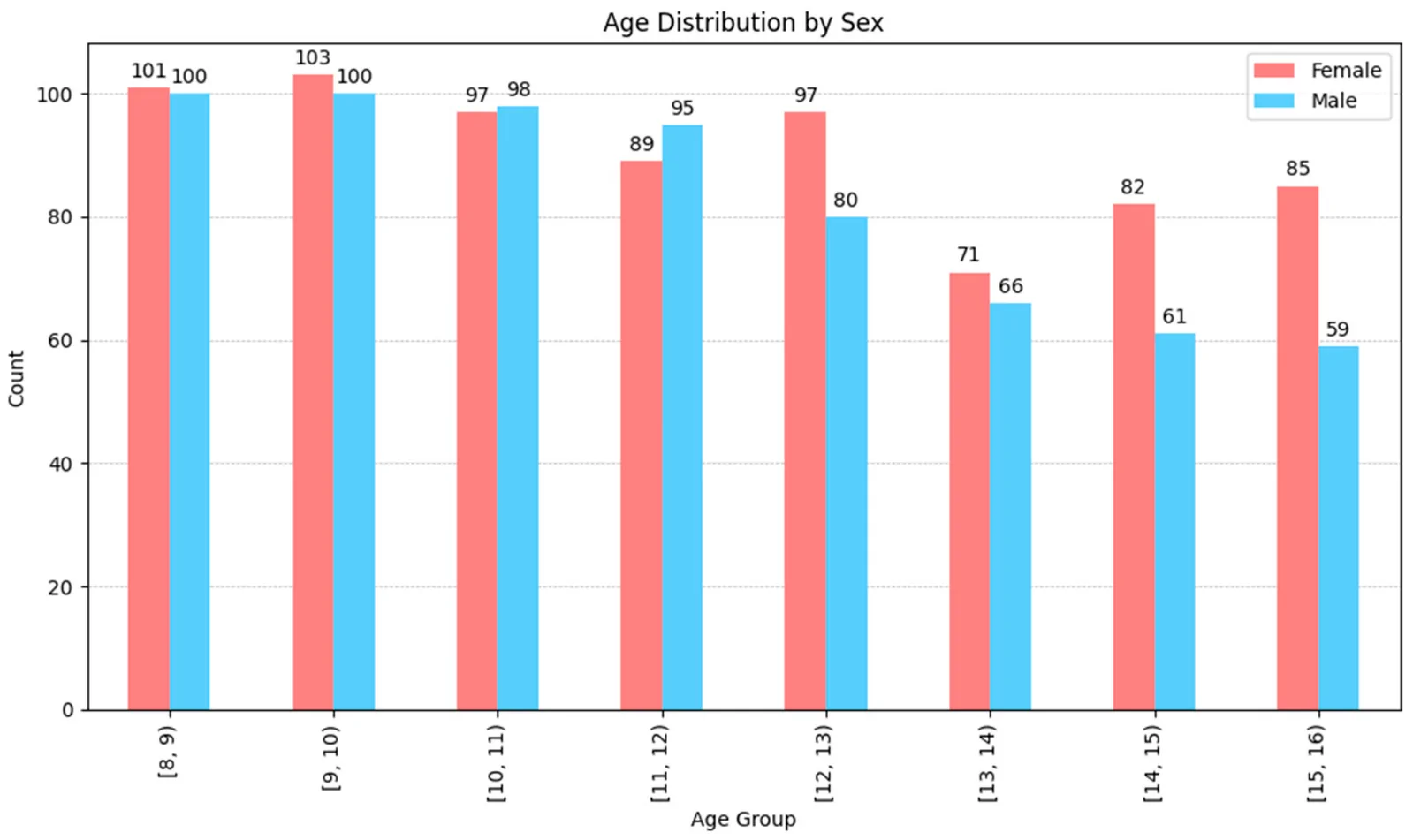
Age and sex distribution by age groups.

**Figure 3:**
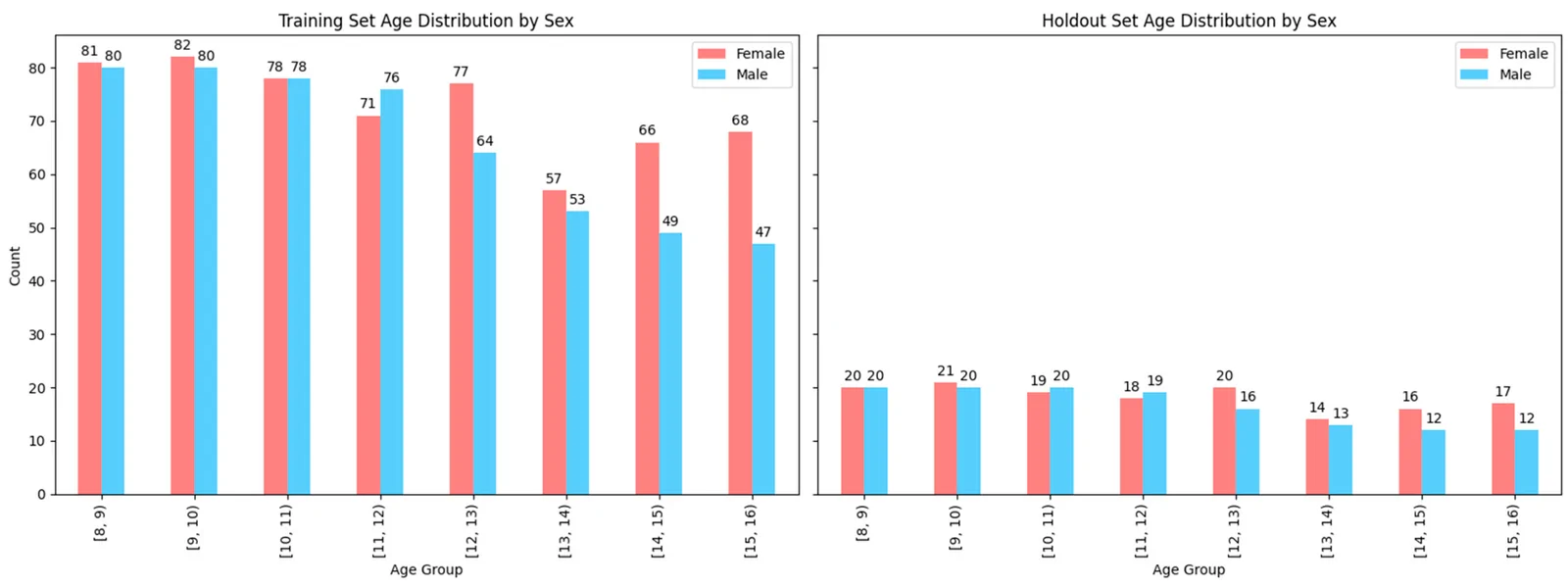
Age and sex distribution of the training and internal holdout sets. Bars indicate the number of female and male participants within each one-year age category.

A total of 1384 panoramic radiographs were included in the analysis. The mean age was 11.79 ± 2.33 years for the 725 females and 11.56 ± 2.24 years for the 659 males. The overall mean age was 11.68 ± 2.29 years, with an age range of 8.00–15.99 years. The age and sex distribution of the dataset is presented in Figure 2. The training set comprised 1107 individuals (580 females and 527 males), whereas the internal holdout set comprised 277 individuals (145 females and 132 males). The age- and sex-specific distributions of both subsets are presented in Figure 3.

### 3.2. Observer Agreement

Observer reliability was assessed using 60 randomly selected panoramic radiographs, corresponding to 420 tooth-stage observations. Linearly weighted Cohen’s kappa was 0.961 (95% CI: 0.943–0.977) for intraobserver agreement and 0.861 (95% CI: 0.834–0.886) for interobserver agreement.

### 3.3. Model Performances

**Figure 4:**
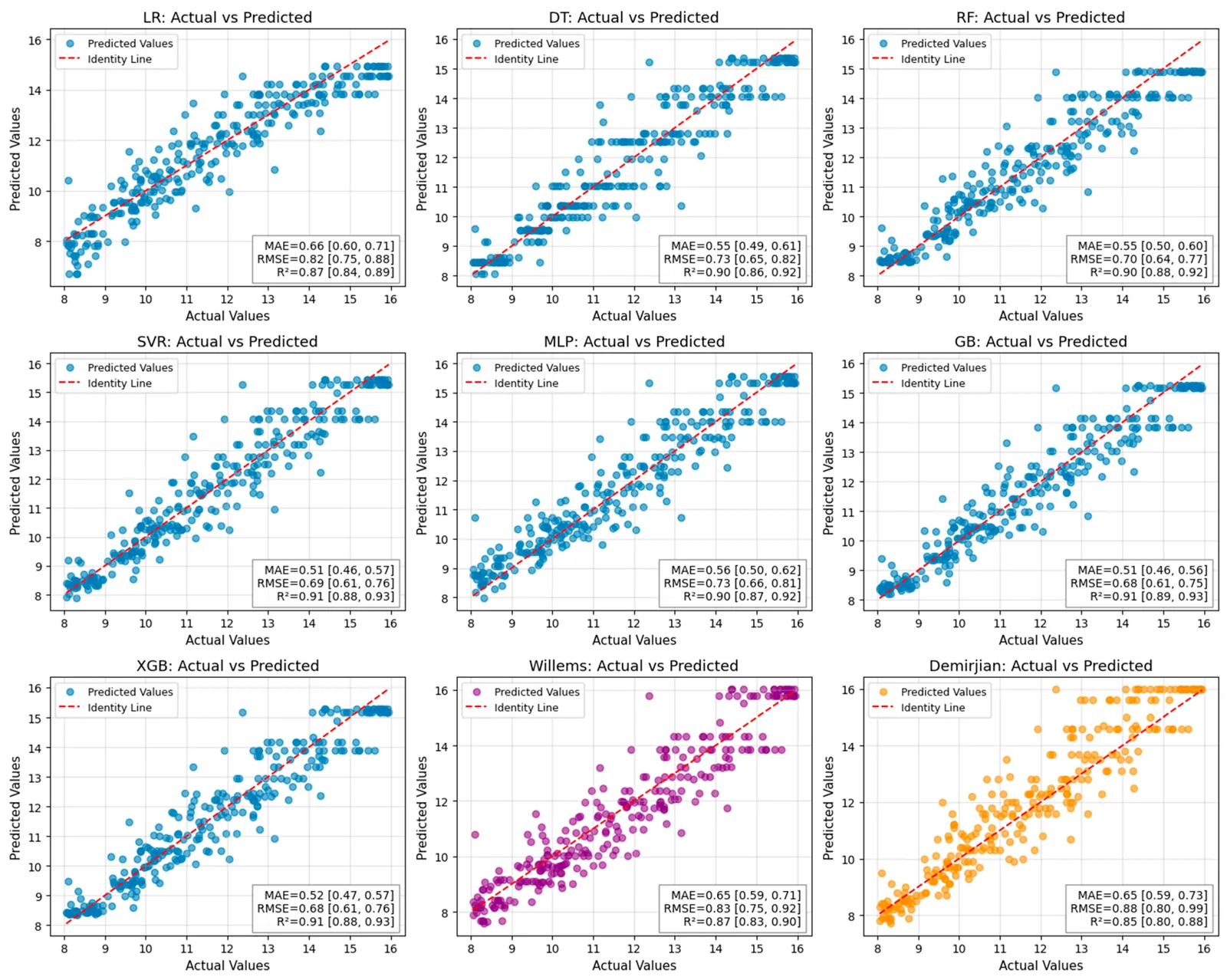
Scatter plots of actual versus predicted age for all models, with the corresponding MAE, RMSE, and R^2^ values and their 95% confidence intervals.

The out-of-fold and holdout performance metrics of the prediction models are presented in Table 1, while the distribution of actual and predicted ages in the holdout set is shown in Figure 4. In the out-of-fold analysis, MAE values for the ML models ranged from 0.544 to 0.655 years. The lowest MAE was obtained with XGBoost Regressor at 0.544 years, followed by Gradient Boosting Regressor and Support Vector Regression, both at 0.546 years. Among the conventional methods, the MAE was 0.639 years for the Willems method and 0.725 years for the Demirjian method. RMSE values ranged from 0.714 to 0.820 years for the ML models, compared with 0.809 years for the Willems method and 0.960 years for the Demirjian method.

In the holdout analysis, MAE values for the ML models ranged from 0.512 to 0.656 years. The lowest MAE was observed for Support Vector Regression and Gradient Boosting Regressor, both at 0.512 years. The MAE was 0.519 years for XGBoost Regressor, 0.546 years for both Random Forest Regressor and Decision Tree Regressor, 0.558 years for MLP Regressor, and 0.656 years for Linear Regression. In the holdout set, the Willems and Demirjian methods yielded MAE values of 0.649 and 0.654 years, respectively. RMSE values ranged from 0.680 to 0.817 years for the ML models and were 0.832 and 0.887 years for the Willems and Demirjian methods, respectively.

Regarding mean error, the Demirjian method showed a statistically significant positive ME in the out-of-fold analysis based on the 95% bootstrap confidence interval (ME = 0.344 years). For the other methods, ME values ranged from −0.017 to 0.038 years. In the holdout analysis, ME values ranged from −0.146 to 0.102 years. Negative ME values were observed for Linear Regression, Random Forest Regressor, Support Vector Regression, Gradient Boosting Regressor, XGBoost Regressor, and the Willems method, whereas positive ME values were obtained for MLP Regressor and the Demirjian method. According to the 95% bootstrap confidence intervals, the Demirjian method showed statistically significant age overestimation, while Linear Regression and the Willems method showed statistically significant age underestimation.

**Figure 5:**
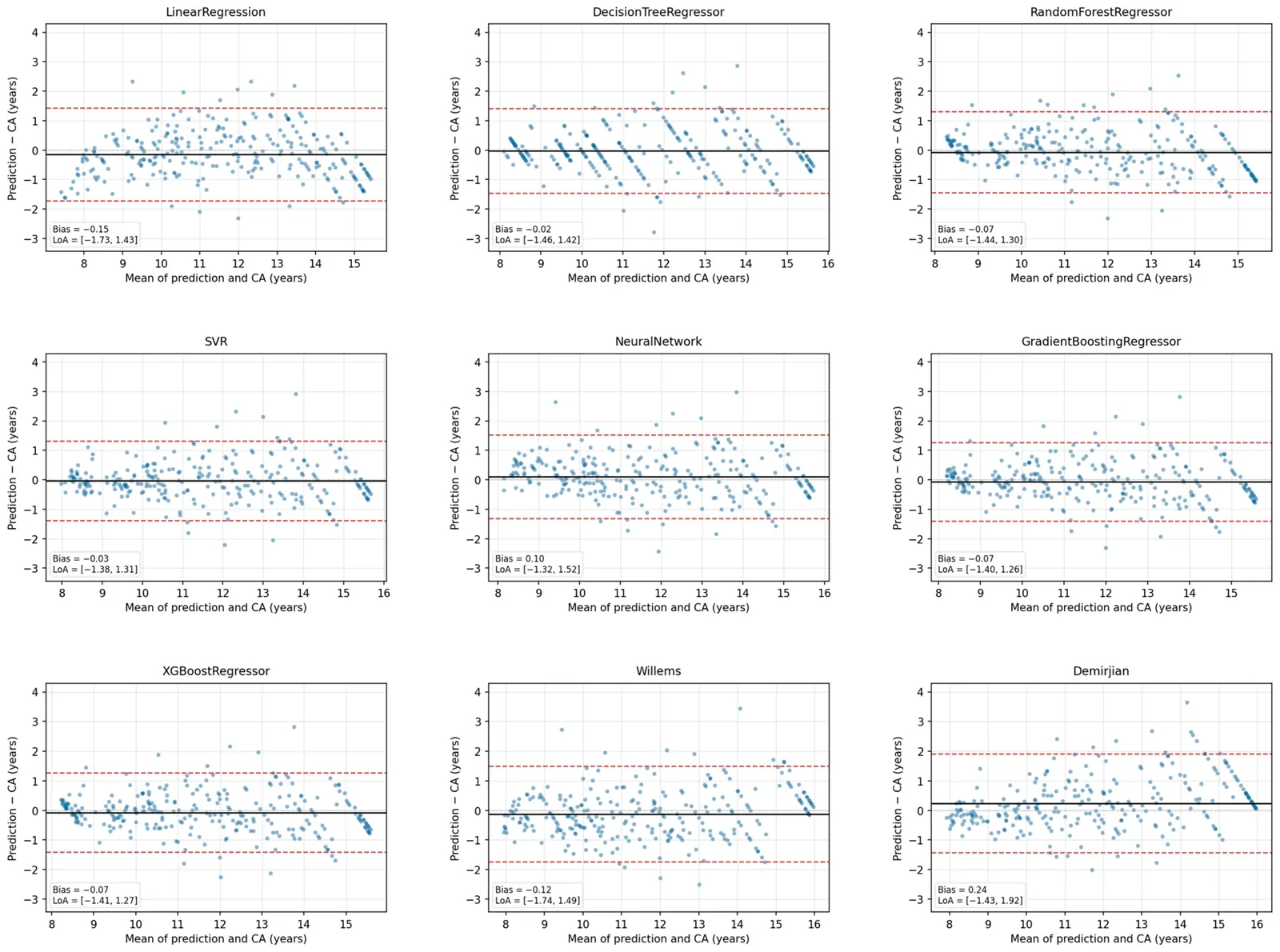
Bland–Altman plots for all methods, internal holdout set. Solid line = bias; dashed lines = 95% limits of agreement.

Bland–Altman analysis of the internal holdout set (Figure 5) showed biases ranging from −0.15 years (LR) to 0.24 years (Demirjian), with 95% limits of agreement spanning approximately ±1.3 to ±1.9 years across methods, narrowest for the gradient-boosting-based regressors and widest for Willems and Demirjian. Extended analyses—including out-of-fold Bland–Altman plots, overall regression-calibration plots with 95% confidence and prediction bands, age-specific mean-error and residual-dispersion analyses, and age-stratified threshold-classification profiles at the 12- and 15-year legal thresholds—are provided in Supplementary File S2.

### 3.4. Comparison of ML Models with Conventional Methods

**Figure 6:**
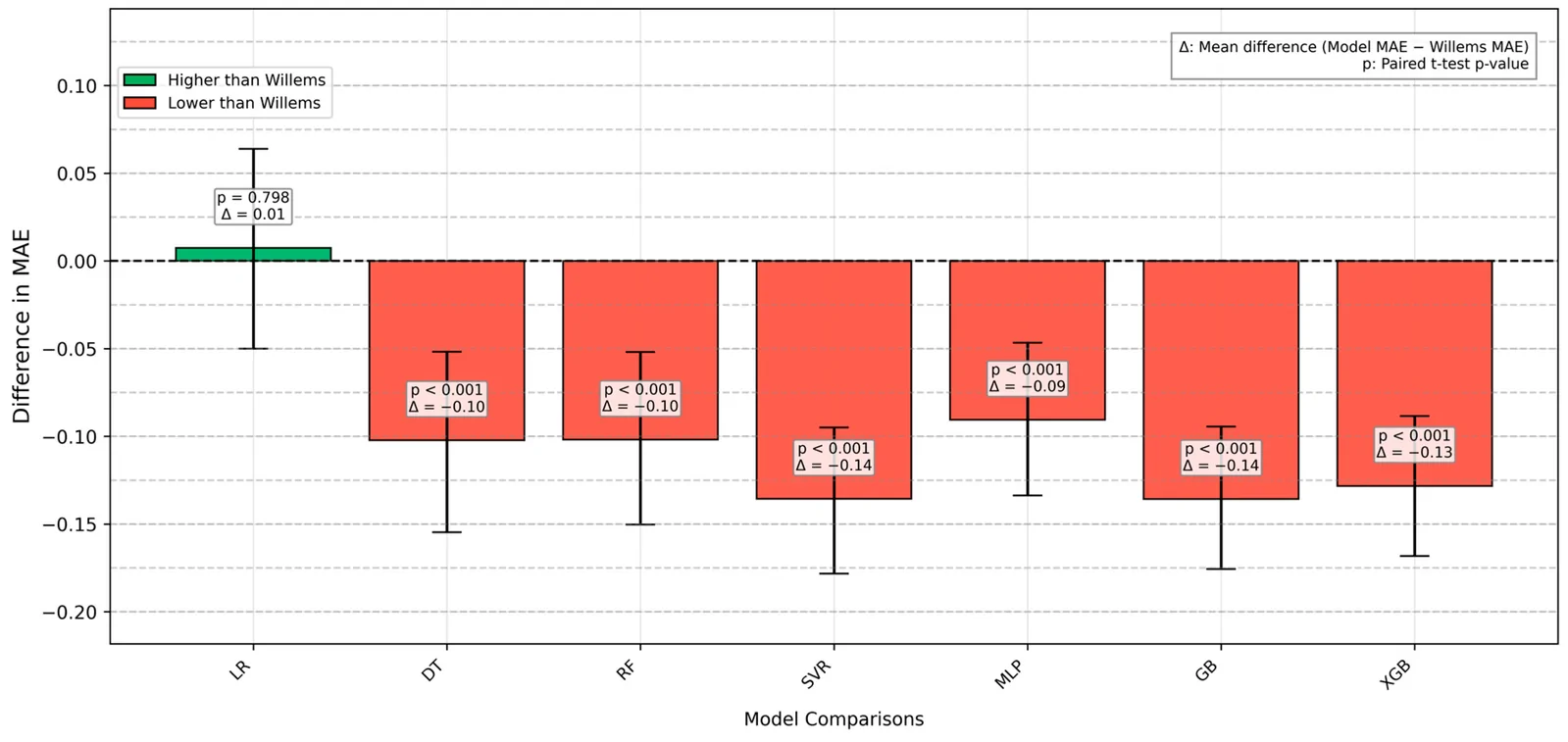
Comparisons of MAE (mean absolute error) between the models and the Willems method, with 95% confidence intervals.

Paired differences in MAE between the Willems method and the ML models were evaluated separately in the holdout analysis (Figure 6). In the holdout set, positive ΔMAE values relative to the Willems method were obtained for all ML models except Linear Regression. The ΔMAE values were 0.10 years for Decision Tree Regressor, 0.10 years for Random Forest Regressor, 0.14 years for Support Vector Regression, 0.09 years for MLP Regressor, 0.14 years for Gradient Boosting Regressor, and 0.13 years for XGBoost Regressor.

**Figure 7:**
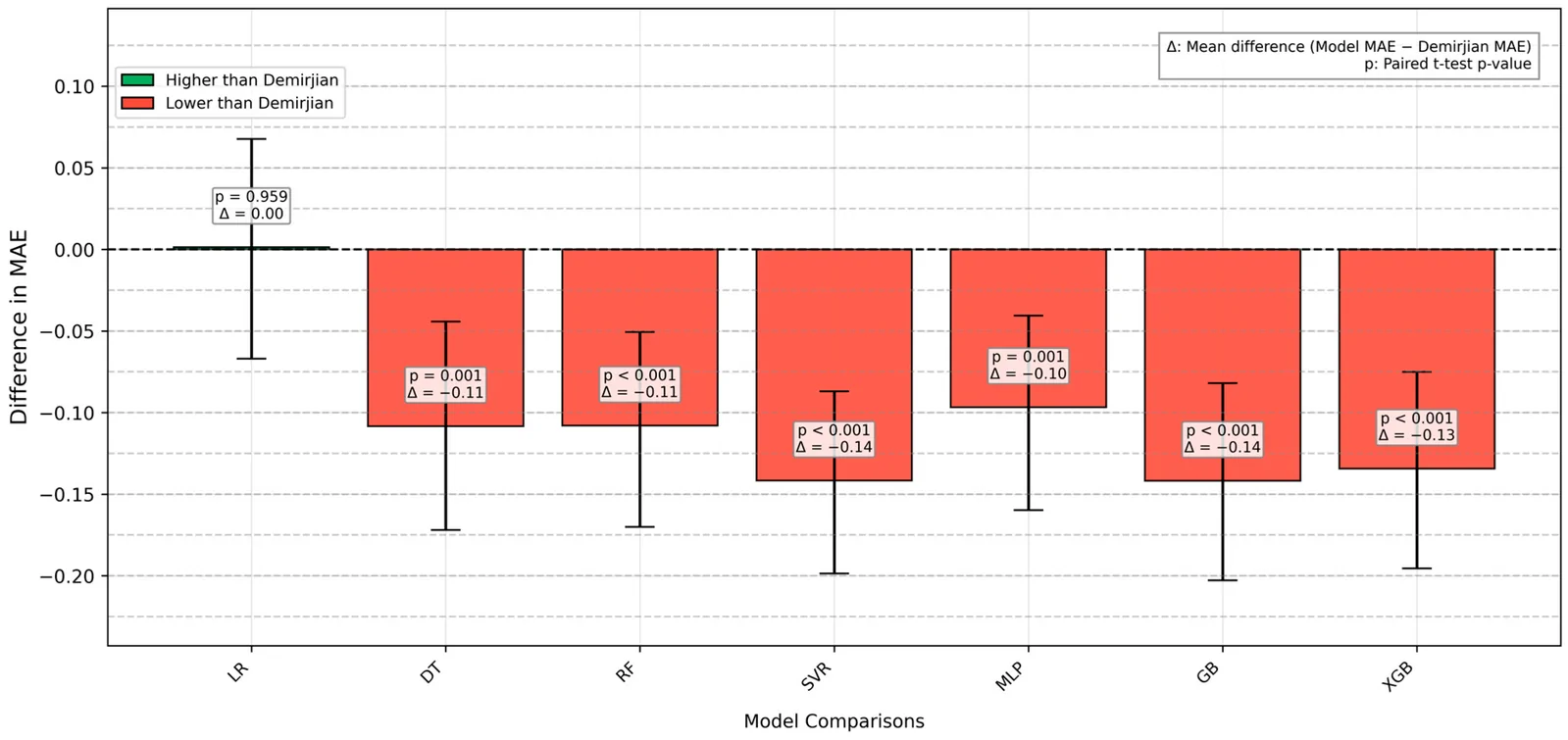
Comparisons of MAE (mean absolute error) between the models and the Demirjian method, with 95% confidence intervals.

In the comparison with the Demirjian method, positive ΔMAE values were likewise obtained for all ML models except Linear Regression in the holdout analysis. The ΔMAE values ranged from 0.00 to 0.14 years (Figure 7).

### 3.5. Analysis by Age Groups

Examination of the holdout predictions showed that mean error values varied across age groups. In general, some models produced positive ME values in the younger age groups, whereas negative ME values were observed for several models in the older age groups.

**Figure 8:**
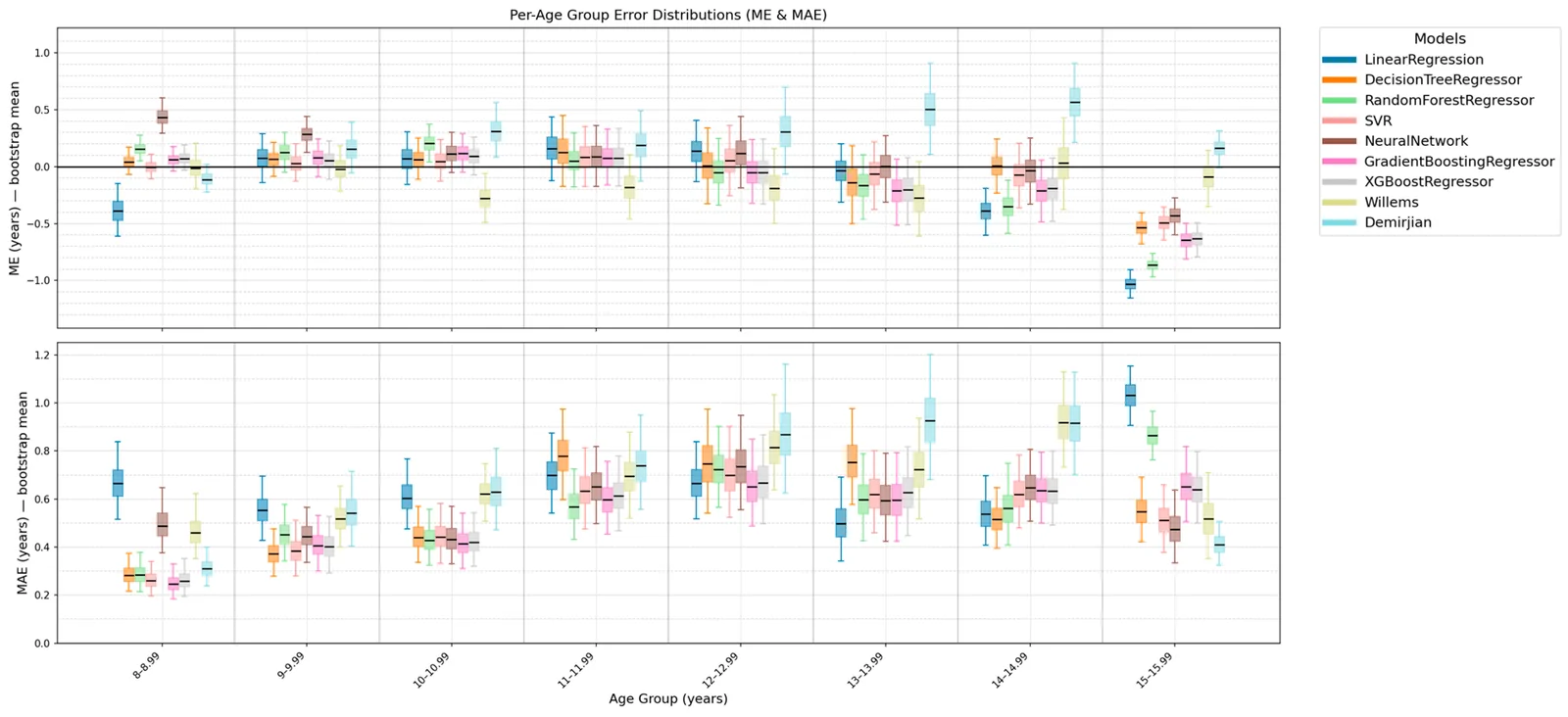
Box plots of mean error (ME) and mean absolute error (MAE) values by age group in the holdout set. The box plots show the distribution of the metrics, and the whiskers represent the 95% confidence intervals (CIs) of the predictions. For each metric, methods closer to the zero reference line indicate better performance. For mean error (ME), positive values (above 0) indicate overestimation, whereas negative values indicate underestimation.

Linear Regression showed a statistically significant negative mean error in the 8.0–8.9-year age group and pronounced negative prediction errors in the 14.0–15.9-year age groups. Random Forest Regressor exhibited the conventional pattern of systematic prediction bias, producing statistically significant overestimation in the 8.0–8.9-year group and statistically significant underestimation in the 15.0–15.9-year group. The MLP Regressor showed a similar pattern to Random Forest Regressor, but with more pronounced systematic bias. All ML models produced statistically significant negative mean errors in the 15.0–15.9-year age group (Figure 8).

Among the conventional methods, the Willems method showed a more balanced mean-error pattern across age groups, whereas the Demirjian method generally tended to overestimate age. Age-group-specific MAE values also varied across models and age groups. Model-specific ME and MAE values, together with their confidence intervals, are presented for each age group in Figure 8.

### 3.6. Threshold-Specific Forensic Performance at the 12-Year Threshold

Out-of-fold and holdout performance results for the 12-year threshold are presented in Table 2a. In the out-of-fold analysis, 481 individuals were aged ≥12 years and 625 were aged <12 years. For the Willems method, sensitivity was 0.82, specificity was 0.95, and balanced accuracy was 0.88. For the Demirjian method, the corresponding values were 0.93, 0.88, and 0.90. Across the ML models, sensitivity ranged from 0.88 to 0.94, specificity from 0.89 to 0.92, and balanced accuracy from 0.90 to 0.92.

In the out-of-fold analysis, LR+ values were 14.89 for the Willems method and 7.37 for the Demirjian method, while those of the ML models ranged from 8.81 to 10.47. LRbelow values were 5.27 for the Willems method and 11.55 for the Demirjian method, compared with 7.42–13.68 across the ML models.

In the holdout analysis, 120 individuals were aged ≥12 years and 157 were aged <12 years. For the Willems method, sensitivity was 0.82, specificity was 0.96, and balanced accuracy was 0.89; for the Demirjian method, the corresponding values were 0.93, 0.87, and 0.90. Across the ML models, sensitivity ranged from 0.91 to 0.93, specificity from 0.91 to 0.94, and balanced accuracy from 0.91 to 0.92. In the holdout analysis, LR+ values for the ML models ranged from 9.86 to 13.62, whereas LRbelow values ranged from 9.56 to 11.57.

### 3.7. Exploratory Threshold-Specific Performance at the 15-Year Threshold

Because the ≥15-year group comprised only individuals aged 15.00–15.99 years, the following 15-year threshold results should be interpreted as exploratory and as reflecting performance near the upper boundary of the study age range. Out-of-fold and holdout performance results for the 15-year threshold are presented in Table 2b. In the out-of-fold analysis, 115 individuals were aged ≥15 years and 992 were aged <15 years. For the Willems method, sensitivity was 0.86, specificity was 0.96, and balanced accuracy was 0.91; for the Demirjian method, the corresponding values were 0.95, 0.91, and 0.93. Linear Regression and Random Forest generated no positive predictions at the 15-year threshold, resulting in a sensitivity of 0.00, specificity of 1.00, and balanced accuracy of 0.50. For Decision Tree Regressor, Support Vector Regression, Gradient Boosting Regressor, and XGBoost Regressor, sensitivity ranged from 0.85 to 0.86, specificity was 0.96, and balanced accuracy was 0.91. For MLP Regressor, sensitivity was 0.59, specificity was 0.97, and balanced accuracy was 0.78.

In the out-of-fold analysis, LR+ values were 20.02 for the Willems method and 10.23 for the Demirjian method. LR+ could not be estimated for Linear Regression or Random Forest because neither model produced positive predictions. Among the remaining ML models, LR+ values ranged from 19.58 to 22.13. LRbelow values were 6.73 for the Willems method, 16.20 for the Demirjian method, and 2.38–6.72 across the ML models.

In the holdout analysis, 29 individuals were aged ≥15 years and 248 were aged <15 years. For the Willems method, sensitivity was 0.79, specificity was 0.96, and balanced accuracy was 0.87; for the Demirjian method, the corresponding values were 0.83, 0.91, and 0.87. Linear Regression and Random Forest again produced no positive predictions at the 15-year threshold. For Decision Tree Regressor, Support Vector Regression, MLP Regressor, Gradient Boosting Regressor, and XGBoost Regressor, sensitivity was 0.79, specificity was 0.96, and balanced accuracy ranged from 0.87 to 0.88. LR+ values were 16.96 for the Willems method, 8.65 for the Demirjian method, and 16.96–18.58 for the ML models that generated positive predictions.

### 3.8. Threshold-Specific Comparison of ML Models with the Willems Method

Paired bootstrap differences between the ML models and the Willems method at the 12-year threshold are presented in Table 3a. In the out-of-fold analysis, ΔSensitivity was positive for all ML models and ranged from 0.06 to 0.11. In the same analysis, ΔSpecificity was negative for all models and ranged from −0.03 to −0.05. ΔBalanced Accuracy ranged from 0.01 to 0.03. Δlog10LR+ was negative for all models, whereas Δlog10LRbelow was positive.

In the holdout analysis at the 12-year threshold, ΔSensitivity was positive for all ML models and ranged from 0.08 to 0.10. ΔSpecificity ranged from −0.02 to −0.04, while ΔBalanced Accuracy ranged from 0.02 to 0.03. Δlog10LR+ was negative for most models, whereas Δlog10LRbelow was positive for all models.

Paired bootstrap differences between the ML models and the Willems method at the 15-year threshold are presented in Table 3b. For Linear Regression and Random Forest, the out-of-fold differences were −0.86 for ΔSensitivity, −0.41 for ΔBalanced Accuracy, and −0.83 for Δlog10LRbelow. In the holdout analysis, the corresponding values were −0.79, −0.37, and −0.64. LR+ could not be estimated for these models because they generated no positive predictions. Decision Tree Regressor, Support Vector Regression, Gradient Boosting Regressor, and XGBoost Regressor showed ΔSensitivity, ΔSpecificity, and ΔBalanced Accuracy values close to those of the Willems method. For MLP Regressor, the out-of-fold differences were −0.27 for ΔSensitivity, 0.02 for ΔSpecificity, and −0.13 for ΔBalanced Accuracy.

### 3.9. Threshold-Specific Comparison of ML Models with the Demirjian Method

Paired bootstrap differences between the ML models and the Demirjian method at the 12-year threshold are presented in Table 3c. In the out-of-fold analysis, ΔSensitivity ranged from −0.05 to 0.01, while ΔSpecificity ranged from 0.02 to 0.04. ΔBalanced Accuracy ranged from approximately 0.00 to 0.01. Δlog10LR+ was positive for all ML models and ranged from 0.08 to 0.15, whereas Δlog10LRbelow ranged from −0.19 to 0.07.

In the holdout analysis at the 12-year threshold, ΔSensitivity ranged from −0.03 to −0.01, ΔSpecificity from 0.04 to 0.06, and ΔBalanced Accuracy from 0.01 to 0.02. Δlog10LR+ ranged from 0.14 to 0.28, whereas Δlog10LRbelow ranged from −0.11 to −0.03.

Paired bootstrap differences between the ML models and the Demirjian method at the 15-year threshold are presented in Table 3d. In the out-of-fold analysis, Linear Regression and Random Forest showed a ΔSensitivity of −0.95, a ΔBalanced Accuracy of approximately −0.43, and a Δlog10LRbelow of −1.21; LR+ could not be estimated for either model because neither generated positive predictions. Among the remaining ML models, ΔSensitivity ranged from −0.36 to −0.09 and ΔSpecificity from 0.05 to 0.07. Δlog10LR+ was positive and ranged from 0.28 to 0.33.

In the holdout analysis, Linear Regression and Random Forest showed a ΔSensitivity of −0.83, a ΔSpecificity of 0.09, and a ΔBalanced Accuracy of −0.37; LR+ again could not be estimated for these models. Among the remaining ML models, ΔSensitivity was approximately −0.03, ΔSpecificity approximately 0.05, and ΔBalanced Accuracy approximately 0.01. Δlog10LR+ ranged from 0.29 to 0.33.

## 4. Discussion

### 4.1. Age Estimation Using the Demirjian and Willems Methods

In the present study, the Demirjian and Willems methods were evaluated as conventional reference approaches, and the findings were interpreted in light of the population-specific differences in performance reported in the literature. In their meta-analysis, Jayaraman et al. (2013) [35] found that the Demirjian method overestimated age by 0.60 years in males and 0.65 years in females [35]. Similarly, Esan et al. (2017) [15] reported age overestimation of 0.62 years in males and 0.72 years in females and concluded that the Demirjian method may not be appropriate for use in populations in which it produces significant systematic overestimation [15]. In studies conducted in Türkiye, Duruk et al. (2022) [36] reported that the Demirjian method overestimated age by 0.68 years and noted that other Turkish studies had documented overestimation ranging from 0.30 to 1.10 years [36].

Studies evaluating age estimation using the Willems method have generally reported lower systematic error. In the meta-analysis by Sehrawat et al. (2017) [37], the method overestimated age by an average of 0.16 years in males and 0.07 years in females [37]. Similarly, Wang et al. (2017) [7] reported mean overestimation of 0.18 years in males and 0.06 years in females [7]. Esan et al. (2017) [15] concluded that although both the Demirjian and Willems methods tended to overestimate age, the Willems method yielded more reliable estimates [15]. In Türkiye, Ozveren et al. (2019) [38] noted that the mean error reported for the Willems method across different studies ranged from an underestimation of 0.062 years to an overestimation of 0.44 years [38].

The findings of the present study are consistent with the tendency toward age overestimation previously reported for the Demirjian method. At the overall sample level, the Demirjian method overestimated age by 0.344 years in the training subset and by 0.242 years in the holdout set. In contrast, the Willems method showed an almost negligible mean error in the training subset (ME = −0.002 years) and underestimated age by 0.122 years in the holdout set. These findings suggest that, in the present sample, the Willems method exhibited a more balanced mean-error pattern than the Demirjian method. Nevertheless, differences in mean error reported for conventional methods may be related to factors such as sample size, age and sex distribution, population-specific patterns of dental maturation, regional variation, nutrition, and socioeconomic conditions [36,39].

### 4.2. Overall Regression Performance of the Machine Learning Models

In terms of overall regression performance, the ML models produced lower error values than the conventional methods, particularly with respect to MAE. In the out-of-fold analysis, the lowest MAE was obtained with XGBoost at 0.544 years, followed by Gradient Boosting and SVR at 0.546 years. By comparison, the MAE was 0.639 years for the Willems method and 0.725 years for the Demirjian method. Among the ML models, the highest MAE was observed for Linear Regression at 0.655 years. In the holdout analysis, the lowest MAE values were again obtained with SVR and Gradient Boosting, both at 0.512 years, followed by XGBoost at 0.519 years. The holdout MAE values for the Willems and Demirjian methods were 0.649 and 0.654 years, respectively, whereas Linear Regression again showed the highest MAE among the ML models at 0.656 years.

Regarding mean error, the Demirjian method produced positive ME values in both the out-of-fold and holdout analyses, at 0.344 and 0.242 years, respectively. This finding supports a tendency toward age overestimation by the Demirjian method in the present sample. The Willems method produced an ME close to zero in the out-of-fold analysis (ME = −0.002 years) and a mildly negative ME in the holdout analysis (ME = −0.122 years). Although ME values were generally lower for the ML models, they varied according to model structure. Linear Regression produced an ME close to zero in the out-of-fold analysis (ME = 0.000 years) but showed a more pronounced negative ME in the holdout analysis (ME = −0.146 years). This may indicate that, despite appearing well balanced in terms of mean error within the training data, the linear model was unable to adequately capture the nonlinear relationship between dental developmental stages and chronological age in the internal holdout set. By contrast, SVR, Gradient Boosting, and XGBoost were distinguished by both lower MAE values and more consistent performance across the overall error metrics.

Similar findings have been reported in studies using dental developmental stages as inputs for ML methods. Galibourg et al. compared several ML regression models based on Demirjian stages with the Demirjian and Willems methods in individuals aged 4–16 years. They reported MAE values of 1.108 years for the Demirjian method, 0.928 years for the Willems method, and 0.729–0.812 years for the ML models [13]. In that study, SVM and RF produced lower error values, whereas Bayesian Ridge Regression and AdaBoost showed more limited performance with higher MAEs. Similarly, in the present study, stage-based ML models generally yielded lower MAEs than the conventional methods, although performance varied by algorithm. SVR, Gradient Boosting, and XGBoost achieved the lowest error values, whereas the MAE of Linear Regression was closer to those of the conventional methods. This finding may reflect the limited ability of a linear model to capture the nonlinear relationship between dental developmental stages and chronological age.

Similarly, Abuabara et al. evaluated eight different ML models using Demirjian stages in a southeastern Brazilian sample and reported an MAE of 0.75 years for both Gradient Boosting and Random Forest [12]. In the same study, the Demirjian method yielded an MAE of 1.34 years, indicating that the ML models produced lower overall error than the conventional approach. This finding is consistent with the present study, in which Gradient Boosting was among the models with the lowest MAE values in both the out-of-fold and holdout analyses. However, whereas Abuabara et al. observed the best performance with Gradient Boosting and Random Forest, SVR, Gradient Boosting, and XGBoost were the leading models in our study. This variation suggests that both algorithm selection and sample characteristics may influence the overall regression performance of stage-based ML models.

Guo et al. evaluated Demirjian stages using SVR, Gradient Boosting, Linear Regression, Random Forest, and Decision Tree models in a large Northern Chinese Han sample aged 5–24 years [14]. SVR showed the best performance in the overall sample and among females, whereas Gradient Boosting performed best among males. The MAE values of the optimal models were 1.246 years in the overall sample, 1.282 years in females, and 1.154 years in males. These findings are consistent with the present study, in which SVR and Gradient Boosting were also among the best-performing models with the lowest MAE values. However, the broader age range and the inclusion of a different set of teeth, including the third molars, in the study by Guo et al. may have contributed to the higher MAE values compared with those observed in our study.

Image-based and hybrid approaches provide a similar perspective on overall regression performance. Simsek et al. applied several regression models after transformer-based feature extraction from panoramic radiographs and reported MAE values of 5.18 months in females and 5.01 months in males using the ExtraTrees model [40]. These results suggest that high-dimensional feature extraction directly from images may capture more detailed age-related information than manually assigned developmental-stage variables. This is consistent with broader evidence from automated image analysis of panoramic radiographs, where deep-learning models applied directly to OPGs have achieved diagnostic agreement exceeding that of human observers (Fleiss’ kappa 0.83 for AI–expert consensus versus 0.71 among human observers) and high accuracy in predicting skeletal maturation directly from radiographic features without relying on discrete, manually assigned staging categories [41]. However, because the present study relied on manually determined Demirjian stages, its predictive performance should be interpreted as being limited by the amount of age-related information contained within those stages.

When considered alongside these previous studies, our findings support the conclusion that stage-based ML models can reduce overall regression error relative to conventional conversion tables. Nevertheless, not all ML algorithms achieved the same level of performance. The higher MAE values observed for Bayesian Ridge Regression and AdaBoost in the study by Galibourg et al. [13], and for Linear Regression in the present study, indicate that ML does not provide a uniform advantage by itself. Rather, predictive performance appears to depend on the underlying model structure, the distributional characteristics of the study sample, and the dental features included as model inputs.

### 4.3. Methodological Challenges of ML Regression Models in Threshold-Specific Age Assessment

Given the large number of paired comparisons performed, findings are interpreted below primarily in terms of the magnitude and consistency of observed differences rather than the binary presence of statistical significance, consistent with the exploratory nature of these threshold-specific analyses. Forensic age estimation in living individuals is important in contexts such as criminal responsibility, victim–offender differentiation, migration and asylum procedures, absence of identity documents, and uncertainty regarding birth records [38]. Under the Turkish Penal Code, the ages of 12 and 15 years constitute key thresholds in the assessment of criminal responsibility in children [33]. Similarly, the minimum age of criminal responsibility is set at 12 years in countries such as the Netherlands and Scotland, and at 15 years in Finland, Sweden, and Denmark [42]. Forensic age estimation of individuals lacking valid identity documentation—including unaccompanied migrant and asylum-seeking minors—is a particularly high-stakes application of this asymmetry, since a threshold misclassification can affect access to child-protection measures or determine criminal-responsibility status [20]. The UN Committee on the Rights of the Child has recommended that no jurisdiction set a minimum age of criminal responsibility below 14 years and has explicitly cautioned against relying on dental or skeletal maturation as the sole basis for age determination [43]. The present findings—where neither the conventional dental methods nor the ML models achieved perfect threshold classification (Table 2a,b)—support treating dental age estimation as one source of probabilistic information within a broader assessment, rather than a stand-alone determinant of legal status [44]. However, legal age thresholds vary among jurisdictions, and the 12- and 15-year cut-offs evaluated in the present study should therefore not be considered universal. Rather, the broader relevance of our approach lies in the threshold-based evaluation framework itself, which can be adapted to different jurisdictions by applying locally relevant legal age limits. Determining whether a child of unknown or disputed age falls below or above these thresholds may therefore have direct forensic and legal consequences. The present study did not address the commonly used 18-year forensic threshold because its design was based on the seven mandibular permanent teeth used in the conventional Demirjian–Willems framework, whereas assessment around 18 years generally requires developmental information extending beyond this tooth set, most notably from the third molars.

The principal finding of the present study is that lower MAE and RMSE values do not necessarily correspond to better forensic threshold-specific classification performance. Although models such as SVR, Gradient Boosting, and XGBoost produced low errors in overall age estimation, their performance at the 12- and 15-year thresholds varied according to model type, age threshold, and the reference method used for comparison. Therefore, the overall regression performance of ML models does not, by itself, represent their decision-making performance at legally relevant age thresholds.

At the 12-year threshold, the ML models showed higher sensitivity than the Willems method. In the out-of-fold analysis, sensitivity was 0.82 for Willems and ranged from 0.88 to 0.94 for the ML models; in the holdout analysis, it was 0.82 for Willems and 0.91–0.93 for the ML models. Paired bootstrap analyses indicated that this increase in sensitivity was statistically significant in favor of ML. However, specificity and LR+ remained higher for the Willems method. In the out-of-fold analysis, Willems achieved a specificity of 0.95 and an LR+ of 14.89, whereas the ML models showed specificities of 0.89–0.92 and LR+ values of 8.81–10.47. Similarly, in the holdout analysis, the LR+ was 17.32 for Willems but ranged from 9.86 to 13.62 for the ML models. Differences in balanced accuracy were more limited: BA values for Willems were 0.88 and 0.89 in the out-of-fold and holdout analyses, respectively, compared with approximately 0.90–0.92 for the ML models. These results indicate that, at the 12-year threshold, the main effect of ML was not an absolute forensic advantage but rather a shift in the sensitivity–specificity trade-off. This trade-off is not ethically neutral. Under the asymmetric framework noted in the Introduction, the higher specificity and LR+ of the Willems method reflect a lower rate of false-positive threshold classifications, whereas the higher sensitivity of the ML models reduces the risk of failing to identify individuals who have genuinely reached the threshold. Which operating point is preferable therefore depends on the specific legal question being addressed (e.g., attribution of criminal responsibility versus eligibility for protective measures), and this should be stated explicitly whenever threshold-specific metrics are used to support a forensic decision.

A different pattern was observed when the ML models were compared with the Demirjian method at the 12-year threshold. In the holdout analysis, Demirjian yielded a sensitivity of 0.93, a specificity of 0.87, a BA of 0.90, and an LR+ of 7.17. By comparison, SVR achieved a sensitivity of 0.91, a specificity of 0.94, a BA of 0.92, and an LR+ of 13.62. Paired bootstrap analyses similarly showed that specificity and Δlog10LR+ were generally significantly in favor of the ML models, whereas differences in sensitivity and BA were more limited. This pattern suggests that the tendency of the Demirjian method to overestimate age may increase sensitivity while simultaneously increasing the number of false-positive classifications, thereby reducing the diagnostic reliability of a positive classification at this threshold.

The 15-year threshold represents a particularly challenging setting for stage-based dental age estimation because it lies close to the upper end of the developmental range represented by the seven mandibular index teeth. Because all methods evaluated here—conventional and ML alike—draw exclusively on the same seven-tooth Demirjian staging information, this ceiling cannot be overcome by better modelling of that information alone. Current recommendations do not find clear evidence that combining multiple dental methods improves accuracy [20], but they do recommend supplementing dental assessment with a non-dental developmental marker once dental maturation approaches completion—most notably clavicular ossification, which continues to mature until approximately 21 years [19]. Extending threshold-specific assessment beyond the resolving limit of the seven-tooth framework would therefore likely require incorporating such a complementary marker rather than further algorithmic refinement of stage-based ML models. The study focused on individuals aged 8.00–15.99 years, consistent with the developmental range targeted by the conventional seven-tooth Demirjian–Willems framework and previous stage-based ML approaches using the same tooth set [8,9,12,13]. However, this design also meant that the ≥15-year category consisted exclusively of individuals aged 15.00–15.99 years and did not represent the broader population aged 15 years and older. Accordingly, the 15-year analysis should be regarded as exploratory. Within this analysis, Linear Regression and Random Forest generated no positive predictions at the 15-year threshold in either the out-of-fold or holdout analyses. Consequently, sensitivity was 0.00, BA was 0.50, and LR+ could not be estimated for these models. The apparent specificity of 1.00 does not represent genuine superiority; rather, it resulted from the models never producing a positive classification. Paired bootstrap analyses likewise showed that ΔSensitivity and ΔBalanced Accuracy for these two models favored the reference methods. Thus, for Linear Regression and Random Forest, the issue at the 15-year threshold was not merely poorer classification performance but the inability to generate a usable positive classification at this threshold.

Although models with better overall regression performance, such as SVR, Gradient Boosting, and XGBoost, were able to generate positive predictions at the 15-year threshold, they did not demonstrate a clear threshold-specific advantage over the Willems method. In the holdout analysis, the Willems method yielded a sensitivity of 0.79, a specificity of 0.96, a BA of 0.87, and an LR+ of 16.96. The corresponding values for these ML models were 0.79 for sensitivity, 0.96 for specificity, approximately 0.88 for BA, and approximately 18.58 for LR+. These numerical differences were small, and the paired bootstrap results did not indicate a practically meaningful advantage in sensitivity, specificity, or BA. In contrast, when compared with the Demirjian method, the ML models produced higher specificity and LR+ values at the 15-year threshold but did not provide an advantage in sensitivity. This pattern may be explained by the tendency of the Demirjian method to overestimate age, which may increase the detection of individuals above the threshold while simultaneously increasing false-positive classifications.

These findings are consistent with the systematic regression bias, age-mimicry effect, and regression-toward-the-mean behavior that may occur in ML regression models [17,18]. When regression models are trained to minimize an overall loss function, predictions at the extremes of the age distribution may be drawn toward the central age range of the dataset. In the present study, the negative ME values produced by the ML models in the oldest age group, 15.00–15.99 years, may reflect this phenomenon and indicate systematic underestimation of chronological age at the upper end of the distribution. This pattern is consistent with the failure of some models to generate positive predictions and with the associated loss of sensitivity at the 15-year threshold. By contrast, at the 12-year threshold, smaller shifts in the prediction distribution may have moved a greater number of individuals into the above-threshold category, thereby increasing sensitivity while reducing specificity and LR+. The fact that the reference methods were developed in different populations, together with the limited ability of dental developmental stages around 12 years of age to discriminate chronological age variation, may also have contributed to the observed threshold-specific performance patterns.

Studies using manually assigned dental developmental stages as inputs for ML models have rarely reported age-group-specific systematic error or threshold-specific performance in detail. Faragalli et al. (2025) [45] and Parlak et al. (2026) [46] proposed Bayesian calibration and Karush–Kuhn–Tucker (KKT)-based optimization approaches to address the systematic bias observed at the extremes of the age distribution in the conventional linear-regression-based Cameriere method. Similar patterns can also be identified in regression-based studies using Demirjian staging. Although Galibourg et al. (2021) [13] did not report age-group-specific mean errors, their scatter plots showed that predictions from the ML regression models were compressed into approximate ranges of 4–15 years and 4–22 years for samples spanning 3–16 years and 3–24 years, respectively. Likewise, the scatter plots presented by Abuabara et al. (2025) [12] suggest that several ML regression models exhibited regression-toward-the-mean patterns at the lower and upper extremes of the age distribution. In the study by Yavuz et al. (2025) [47], which compared deep learning with conventional dental age-estimation methods, an age-dependent centralizing pattern in regression-based deep-learning predictions was explicitly reported [47].

For these reasons, a fundamental limitation of ML regression models is that they are optimized to predict chronological age as a continuous outcome, whereas forensic practice often requires a determination of whether an individual falls below or above a specific legal age threshold. Moreover, in the present study, the ML models were trained and optimized using data from the target population, while the Demirjian and Willems methods were applied as fixed formula-based reference approaches. Despite this apparent advantage, the ML models did not consistently outperform the conventional methods in threshold-specific or likelihood-ratio-based analyses. These findings indicate that improved population-specific regression accuracy does not necessarily translate into improved classification performance at legally relevant age thresholds.

These findings are also consistent with the broader methodological debate surrounding the use of artificial intelligence in dental age estimation. Although interest in AI and ML methods continues to increase, it has been emphasized that many studies do not adequately address issues already recognized in conventional age-estimation research, including sample-distribution effects, age-group-specific bias, threshold performance, and forensic interpretability [17]. Therefore, reporting only overall mean error values or point estimates is insufficient to meet the forensic requirements of age estimation in living individuals when legally or human-rights-relevant thresholds are involved. ML studies in this field should consequently be evaluated not only in terms of overall regression performance but also with respect to systematic bias and threshold-specific decision performance.

### 4.4. Limitations

This study was conducted retrospectively at a single center and therefore represents internal validation only. No external validation using data from a different center, imaging system, or source population was performed; consequently, the generalizability of the findings to other settings and populations remains uncertain. Because an adequate number of cases younger than 8 years was not available at our center, this age group could not be included. The upper age limit was selected to maintain methodological consistency with the seven-tooth Demirjian–Willems framework and comparable stage-based ML studies. However, this necessarily restricted the ≥15-year category to individuals aged 15.00–15.99 years, with no individuals aged 16 years or older. Consequently, the 15-year threshold analysis reflects performance near the upper boundary of the observed age distribution rather than performance in a broadly sampled population aged 15 years and older. The relatively small number of individuals in this category may also have reduced the stability of sensitivity, balanced accuracy, and likelihood-ratio estimates. Therefore, the 15-year findings should be considered exploratory and should not be generalized to broader ≥15-year populations without further validation in samples extending beyond the present age range.

Finally, the ML models did not learn features directly from the radiographic images but generated predictions from manually assigned Demirjian developmental stages and sex. Model performance was therefore limited by the biological information regarding chronological age contained in these dental stages. Future studies should use larger, multicenter samples with external validation and should evaluate additional age thresholds and measures of individual-level uncertainty.

## 5. Conclusions

In this study, ML regression models, particularly SVR, Gradient Boosting, and XGBoost, produced lower MAE and RMSE values than the Demirjian and Willems methods. However, these improvements in overall regression performance did not translate into a consistent advantage in threshold-specific classification performance. At the 12-year threshold, the ML models increased sensitivity in some comparisons but showed reduced specificity and LR+ values. In the exploratory 15-year analysis, some models failed to generate positive predictions, whereas others demonstrated classification performance similar to that of the Willems method. These findings indicate that lower mean prediction error alone should not be interpreted as evidence of better performance at legally relevant age thresholds.

In forensic practice, the principal question is often not the exact point estimate of chronological age, but whether an individual falls below or above a legally relevant age threshold. Accordingly, ML-based age-estimation models should be validated not only using overall regression metrics, such as MAE, RMSE, and R^2^, but also in terms of threshold-specific sensitivity, specificity, balanced accuracy, and likelihood ratios, before their implementation in forensic practice. Although ML regression models represent promising tools for dental age estimation, they should be used cautiously at legally consequential age thresholds and incorporated into forensic decision-making only after adequate threshold-level validation. The present findings should therefore be regarded as a baseline evaluation of stage-based ML modelling rather than as a definitive image-based ML solution for dental age estimation.

## Figures and Tables

**Table 1:** Summary of training (out-of-fold) and holdout performance metrics for the regression models.

Model	ME ± SD [95% CI]	MAE ± SD [95% CI]	RMSE ± SD [95% CI]	R^2^ ± SD [95% CI]
**Out-of-fold**				
Linear Regression	0.000 ± 0.025 [−0.049, 0.049]	0.655 ± 0.014 [0.627, 0.683]	0.820 ± 0.017 [0.786, 0.854]	0.872 ± 0.006 [0.858, 0.884]
Decision Tree Regressor	0.028 ± 0.023 [−0.016, 0.074]	0.566 ± 0.015 [0.536, 0.596]	0.764 ± 0.022 [0.721, 0.808]	0.888 ± 0.008 [0.873, 0.903]
Random Forest Regressor	0.000 ± 0.022 [−0.043, 0.044]	0.576 ± 0.014 [0.550, 0.604]	0.745 ± 0.018 [0.708, 0.783]	0.894 ± 0.006 [0.882, 0.906]
Support Vector Regression	0.038 ± 0.022 [−0.004, 0.083]	0.546 ± 0.014 [0.519, 0.574]	0.729 ± 0.020 [0.687, 0.771]	0.899 ± 0.007 [0.884, 0.911]
MLP Regressor	−0.017 ± 0.024 [−0.061, 0.028]	0.605 ± 0.014 [0.578, 0.634]	0.776 ± 0.018 [0.742, 0.813]	0.885 ± 0.006 [0.872, 0.896]
Gradient Boosting Regressor	0.001 ± 0.022 [−0.040, 0.044]	0.546 ± 0.014 [0.521, 0.574]	0.717 ± 0.019 [0.679, 0.757]	0.902 ± 0.006 [0.889, 0.913]
XGBoost Regressor	0.000 ± 0.022 [−0.041, 0.042]	0.544 ± 0.014 [0.518, 0.571]	0.714 ± 0.019 [0.677, 0.754]	0.902 ± 0.006 [0.890, 0.914]
Willems *	−0.002 ± 0.024 [−0.049, 0.047]	0.639 ± 0.015 [0.608, 0.669]	0.809 ± 0.020 [0.770, 0.850]	0.875 ± 0.008 [0.859, 0.888]
Demirjian *	0.344 ± 0.027 [0.292, 0.396]	0.725 ± 0.019 [0.690, 0.763]	0.960 ± 0.027 [0.911, 1.013]	0.824 ± 0.012 [0.798, 0.845]
**Internal holdout**				
Linear Regression	−0.146 ± 0.048 [−0.238, −0.054]	0.656 ± 0.030 [0.597, 0.715]	0.817 ± 0.036 [0.745, 0.886]	0.872 ± 0.013 [0.842, 0.895]
Decision Tree Regressor	−0.023 ± 0.044 [−0.110, 0.058]	0.546 ± 0.030 [0.491, 0.607]	0.734 ± 0.043 [0.653, 0.816]	0.897 ± 0.015 [0.864, 0.922]
Random Forest Regressor	−0.070 ± 0.042 [−0.154, 0.005]	0.546 ± 0.027 [0.496, 0.599]	0.702 ± 0.036 [0.633, 0.771]	0.905 ± 0.012 [0.880, 0.925]
Support Vector Regression	−0.034 ± 0.041 [−0.115, 0.035]	0.512 ± 0.028 [0.458, 0.566]	0.687 ± 0.040 [0.605, 0.766]	0.909 ± 0.013 [0.880, 0.933]
MLP Regressor	0.102 ± 0.042 [0.014, 0.178]	0.558 ± 0.029 [0.502, 0.615]	0.732 ± 0.042 [0.652, 0.811]	0.897 ± 0.014 [0.867, 0.921]
Gradient Boosting Regressor	−0.067 ± 0.040 [−0.146, 0.003]	0.512 ± 0.027 [0.462, 0.565]	0.680 ± 0.038 [0.607, 0.755]	0.911 ± 0.012 [0.884, 0.931]
XGBoost Regressor	−0.069 ± 0.040 [−0.148, 0.002]	0.519 ± 0.027 [0.470, 0.574]	0.688 ± 0.039 [0.612, 0.763]	0.909 ± 0.012 [0.881, 0.930]
Willems	−0.122 ± 0.048 [−0.219, −0.032]	0.649 ± 0.031 [0.593, 0.712]	0.832 ± 0.042 [0.753, 0.918]	0.867 ± 0.017 [0.829, 0.895]
Demirjian	0.242 ± 0.051 [0.135, 0.336]	0.654 ± 0.037 [0.582, 0.727]	0.887 ± 0.050 [0.784, 0.980]	0.849 ± 0.021 [0.803, 0.887]

ME: mean error; MAE: mean absolute error; RMSE: rooted mean squared error; R^2^: coefficient of determination; CI: confidence interval; SD: standard deviation. * For Willems and Demirjian, OOF denotes performance calculated on the same training subset used for out-of-fold ML predictions; these methods were not trained and therefore do not produce out-of-fold predictions in the model-training sense. Bold subheadings (“Out-of-fold” and “Internal holdout”) identify the evaluation set to which the results in the rows below refer.

**Table 2:** (**a**) Threshold-specific forensic performance for age at least 12 years in out-of-fold and holdout analyses. (**b**) Exploratory threshold-specific performance at the 15-year threshold in out-of-fold and holdout analyses.

(**a**)												
**Split**	**Method**	**True + (n)**	**TP**	**FN**	**FP**	**TN**	Sens. [95% CI] *	Spec. [95% CI] *	**BA [95% CI]**	**LR+ [95% CI]**	**LR− [95% CI]**	**LR_below [95% CI]**
OOF	Willems	481	395	86	34	591	0.82 [0.78, 0.85]	0.95 [0.92, 0.96]	0.88 [0.86, 0.90]	14.89 [11.14, 21.19]	0.19 [0.15, 0.23]	5.27 [4.42, 6.51]
OOF	Demirjian	481	445	36	78	547	0.93 [0.90, 0.95]	0.88 [0.85, 0.90]	0.90 [0.88, 0.92]	7.37 [6.00, 9.14]	0.09 [0.06, 0.11]	11.55 [8.74, 16.24]
OOF	Linear regression	481	450	31	66	560	0.94 [0.91, 0.96]	0.89 [0.87, 0.92]	0.92 [0.90, 0.93]	8.81 [7.15, 11.43]	0.07 [0.05, 0.10]	13.68 [10.34, 20.26]
OOF	Decision tree	481	438	43	56	570	0.91 [0.88, 0.93]	0.91 [0.89, 0.93]	0.91 [0.89, 0.93]	10.10 [8.11, 13.33]	0.10 [0.07, 0.13]	10.08 [7.80, 14.29]
OOF	Random forest	481	442	39	56	570	0.92 [0.89, 0.94]	0.91 [0.89, 0.93]	0.91 [0.90, 0.93]	10.19 [8.13, 13.42]	0.09 [0.06, 0.12]	11.10 [8.55, 15.77]
OOF	SVR	481	441	40	56	570	0.92 [0.89, 0.94]	0.91 [0.89, 0.93]	0.91 [0.90, 0.93]	10.16 [8.07, 13.22]	0.09 [0.07, 0.12]	10.83 [8.23, 14.81]
OOF	MLP	481	422	59	52	574	0.88 [0.84, 0.91]	0.92 [0.89, 0.94]	0.90 [0.88, 0.92]	10.47 [8.15, 13.77]	0.13 [0.10, 0.17]	7.42 [5.87, 9.60]
OOF	Gradient boosting	481	439	42	56	570	0.91 [0.88, 0.94]	0.91 [0.89, 0.93]	0.91 [0.89, 0.93]	10.12 [7.99, 13.27]	0.10 [0.07, 0.13]	10.32 [7.87, 14.22]
OOF	XGBoost	481	441	40	57	569	0.92 [0.89, 0.94]	0.91 [0.88, 0.93]	0.91 [0.90, 0.93]	9.99 [8.03, 13.59]	0.09 [0.07, 0.12]	10.81 [8.39, 15.06]
Holdout	Willems	120	99	21	7	150	0.82 [0.75, 0.89]	0.96 [0.91, 0.98]	0.89 [0.85, 0.93]	17.32 [9.81, 41.34]	0.19 [0.12, 0.26]	5.36 [3.89, 8.46]
Holdout	Demirjian	120	112	8	20	137	0.93 [0.87, 0.97]	0.87 [0.81, 0.92]	0.90 [0.87, 0.94]	7.17 [5.13, 11.67]	0.08 [0.03, 0.13]	12.39 [7.53, 29.62]
Holdout	Linear regression	120	111	9	14	143	0.93 [0.86, 0.97]	0.91 [0.85, 0.95]	0.92 [0.89, 0.95]	10.04 [6.65, 18.04]	0.09 [0.04, 0.14]	11.57 [7.17, 27.22]
Holdout	Decision tree	120	109	11	14	143	0.91 [0.84, 0.95]	0.91 [0.85, 0.95]	0.91 [0.88, 0.94]	9.86 [6.67, 16.94]	0.10 [0.05, 0.17]	9.56 [5.98, 19.85]
Holdout	Random forest	120	109	11	13	144	0.91 [0.84, 0.95]	0.92 [0.86, 0.96]	0.91 [0.88, 0.95]	10.59 [6.94, 19.59]	0.10 [0.05, 0.17]	9.62 [5.93, 19.35]
Holdout	SVR	120	109	11	10	147	0.91 [0.84, 0.95]	0.94 [0.89, 0.97]	0.92 [0.89, 0.95]	13.62 [8.52, 32.17]	0.10 [0.05, 0.16]	9.82 [6.11, 20.10]
Holdout	MLP	120	110	10	12	145	0.92 [0.85, 0.96]	0.92 [0.87, 0.96]	0.92 [0.89, 0.95]	11.54 [7.58, 23.27]	0.09 [0.05, 0.15]	10.61 [6.58, 21.80]
Holdout	Gradient boosting	120	109	11	12	145	0.91 [0.84, 0.95]	0.92 [0.87, 0.96]	0.92 [0.88, 0.95]	11.44 [7.37, 23.37]	0.10 [0.05, 0.16]	9.69 [6.30, 19.68]
Holdout	XGBoost	120	110	10	12	145	0.92 [0.85, 0.96]	0.92 [0.87, 0.96]	0.92 [0.89, 0.95]	11.54 [7.54, 22.82]	0.09 [0.04, 0.15]	10.61 [6.66, 24.80]
(**b**)												
**Split**	**Method**	**True + (n)**	**TP**	**FN**	**FP**	**TN**	Sens. [95% CI] *	Spec. [95% CI] *	**BA [95% CI]**	**LR+ [95% CI]**	**LR− [95% CI]**	**LR_below [95% CI]**
OOF	Willems	115	99	16	42	949	0.86 [0.78, 0.92]	0.96 [0.94, 0.97]	0.91 [0.88, 0.94]	20.02 [15.18, 27.60]	0.15 [0.09, 0.22]	6.73 [4.60, 11.64]
OOF	Demirjian	115	109	6	91	900	0.95 [0.89, 0.98]	0.91 [0.89, 0.93]	0.93 [0.91, 0.95]	10.23 [8.54, 12.62]	0.06 [0.02, 0.11]	16.20 [9.02, 42.55]
OOF	Linear regression	115	0	115	0	992	0.00 [0.00, 0.03]	1.00 [1.00, 1.00] ‡	0.50 [0.50, 0.50]	NE	1.00 [1.00, 1.00]	1.00 [1.00, 1.00]
OOF	Decision tree	115	99	16	43	949	0.86 [0.78, 0.92]	0.96 [0.94, 0.97]	0.91 [0.88, 0.94]	19.58 [15.18, 27.86]	0.15 [0.09, 0.22]	6.72 [4.62, 11.72]
OOF	Random forest	115	0	115	0	992	0.00 [0.00, 0.03]	1.00 [1.00, 1.00] ‡	0.50 [0.50, 0.50]	NE	1.00 [1.00, 1.00]	1.00 [1.00, 1.00]
OOF	SVR	115	98	17	41	951	0.85 [0.77, 0.91]	0.96 [0.94, 0.97]	0.91 [0.87, 0.94]	20.32 [15.28, 29.68]	0.16 [0.09, 0.23]	6.35 [4.31, 10.78]
OOF	MLP	115	68	47	26	966	0.59 [0.50, 0.68]	0.97 [0.96, 0.98]	0.78 [0.74, 0.83]	22.13 [15.72, 36.58]	0.42 [0.33, 0.51]	2.38 [1.95, 3.04]
OOF	Gradient boosting	115	98	17	41	951	0.85 [0.77, 0.91]	0.96 [0.94, 0.97]	0.91 [0.87, 0.94]	20.32 [15.71, 28.94]	0.16 [0.10, 0.23]	6.35 [4.41, 10.21]
OOF	XGBoost	115	98	17	41	951	0.85 [0.77, 0.91]	0.96 [0.94, 0.97]	0.91 [0.87, 0.94]	20.32 [15.60, 28.78]	0.16 [0.09, 0.23]	6.35 [4.35, 10.78]
Holdout	Willems	29	23	6	11	237	0.79 [0.60, 0.92]	0.96 [0.92, 0.98]	0.87 [0.80, 0.95]	16.96 [9.98, 37.09]	0.23 [0.09, 0.40]	4.40 [2.49, 11.40]
Holdout	Demirjian	29	24	5	23	225	0.83 [0.64, 0.94]	0.91 [0.86, 0.94]	0.87 [0.80, 0.94]	8.65 [6.06, 14.02]	0.20 [0.07, 0.35]	4.94 [2.87, 15.07]
Holdout	Linear regression	29	0	29	0	248	0.00 [0.00, 0.12]	1.00 [0.99, 1.00] ‡	0.50 [0.50, 0.50]	NE	0.99 [0.98, 0.99]	1.01 [1.01, 1.02]
Holdout	Decision tree	29	23	6	11	237	0.79 [0.60, 0.92]	0.96 [0.92, 0.98]	0.87 [0.80, 0.95]	16.96 [10.34, 35.30]	0.23 [0.09, 0.39]	4.40 [2.59, 11.68]
Holdout	Random forest	29	0	29	0	248	0.00 [0.00, 0.12]	1.00 [0.99, 1.00] ‡	0.50 [0.50, 0.50]	NE	0.99 [0.98, 0.99]	1.01 [1.01, 1.02]
Holdout	SVR	29	23	6	10	238	0.79 [0.60, 0.92]	0.96 [0.93, 0.98]	0.88 [0.80, 0.95]	18.58 [10.74, 39.87]	0.23 [0.08, 0.37]	4.42 [2.67, 11.97]
Holdout	MLP	29	23	6	10	238	0.79 [0.60, 0.92]	0.96 [0.93, 0.98]	0.88 [0.80, 0.95]	18.58 [10.68, 43.77]	0.23 [0.08, 0.39]	4.42 [2.58, 11.98]
Holdout	Gradient boosting	29	23	6	10	238	0.79 [0.60, 0.92]	0.96 [0.93, 0.98]	0.88 [0.80, 0.95]	18.58 [10.92, 42.45]	0.23 [0.10, 0.38]	4.42 [2.62, 10.47]
Holdout	XGBoost	29	23	6	10	238	0.79 [0.60, 0.92]	0.96 [0.93, 0.98]	0.88 [0.80, 0.95]	18.58 [11.16, 40.03]	0.23 [0.10, 0.38]	4.42 [2.63, 10.31]

(**a**) TP: true positive, true age ≥12 and predicted age ≥12; FN: false negative, true age ≥12 and predicted age <12; FP: false positive, true age <12 and predicted age ≥12; TN: true negative, true age <12 and predicted age <12; Sens.: sensitivity; Spec.: specificity; BA: balanced accuracy; LR+: likelihood ratio for a positive finding; LR−: likelihood ratio for a negative finding; LR_below: reciprocal of LR−. True + (n): number of individuals in the true at-or-above-threshold group (TP + FN). Values are presented as point estimate [95% CI]. * Sensitivity and specificity CIs are exact binomial (Clopper–Pearson) intervals. Balanced-accuracy CIs are based on the variance of the two class-specific proportions. Likelihood-ratio CIs are taken from the threshold-analysis output. (**b**) TP: true positive, true age ≥15 and predicted age ≥15; FN: false negative, true age ≥15 and predicted age <15; FP: false positive, true age <15 and predicted age ≥15; TN: true negative, true age <15 and predicted age <15; Sens.: sensitivity; Spec.: specificity; BA: balanced accuracy; LR+: likelihood ratio for a positive finding; LR−: likelihood ratio for a negative finding; LR_below: reciprocal of LR−. True + (n): number of individuals in the true at-or-above-threshold group (TP + FN). Values are presented as point estimate [95% CI]. * Sensitivity and specificity CIs are exact binomial (Clopper–Pearson) intervals. Balanced-accuracy CIs are based on the variance of the two class-specific proportions. Likelihood-ratio CIs are taken from the threshold-analysis output. NE: not estimable because the model produced no positive predictions at the 15-year threshold. ‡ No positive predictions at the 15-year threshold; high specificity should not be interpreted as superior threshold performance. The ≥15-year category comprised individuals aged 15.00–15.99 years only; therefore, the 15-year analysis should be interpreted as exploratory.

**Table 3:** (**a**) Paired bootstrap differences in threshold-specific forensic performance between machine learning models and the Willems method at the 12-year threshold. (**b**) Paired bootstrap differences in exploratory threshold-specific performance between machine learning models and the Willems method at the 15-year threshold. (**c**) Paired bootstrap differences in threshold-specific forensic performance between machine learning models and the Demirjian method at the 12-year threshold. (**d**) Paired bootstrap differences in exploratory threshold-specific performance between machine learning models and the Demirjian method at the 15-year threshold.

(**a**)						
**Split**	**ML Model**	**ΔSens.**	**ΔSpec.**	**ΔBA**	**Δlog10LR+**	**Δlog10LR_below**
OOF	Linear regression	0.11 [0.09, 0.14] *	−0.05 [−0.07, −0.03] †	0.03 [0.02, 0.05] *	−0.23 [−0.35, −0.13] †	0.41 [0.30, 0.56] *
OOF	Decision tree	0.09 [0.06, 0.12] *	−0.04 [−0.05, −0.02] †	0.03 [0.01, 0.04] *	−0.17 [−0.27, −0.09] †	0.28 [0.19, 0.39] *
OOF	Random forest	0.10 [0.07, 0.12] *	−0.04 [−0.05, −0.02] †	0.03 [0.02, 0.05] *	−0.17 [−0.27, −0.08] †	0.32 [0.23, 0.43] *
OOF	SVR	0.10 [0.07, 0.12] *	−0.04 [−0.05, −0.02] †	0.03 [0.02, 0.05] *	−0.17 [−0.27, −0.08] †	0.31 [0.22, 0.42] *
OOF	MLP	0.06 [0.03, 0.08] *	−0.03 [−0.04, −0.02] †	0.01 [−0.00, 0.03]	−0.15 [−0.26, −0.07] †	0.15 [0.08, 0.23] *
OOF	Gradient boosting	0.09 [0.07, 0.12] *	−0.04 [−0.05, −0.02] †	0.03 [0.01, 0.04] *	−0.17 [−0.27, −0.08] †	0.29 [0.20, 0.40] *
OOF	XGBoost	0.10 [0.07, 0.12] *	−0.04 [−0.05, −0.02] †	0.03 [0.01, 0.05] *	−0.17 [−0.28, −0.09] †	0.31 [0.22, 0.42] *
Holdout	Linear regression	0.10 [0.05, 0.16] *	−0.04 [−0.08, −0.01] †	0.03 [−0.00, 0.06]	−0.24 [−0.56, −0.02] †	0.33 [0.16, 0.60] *
Holdout	Decision tree	0.08 [0.03, 0.14] *	−0.04 [−0.08, −0.01] †	0.02 [−0.01, 0.05]	−0.24 [−0.56, −0.06] †	0.25 [0.09, 0.49] *
Holdout	Random forest	0.08 [0.04, 0.13] *	−0.04 [−0.07, −0.01] †	0.02 [−0.00, 0.05]	−0.21 [−0.51, −0.04] †	0.25 [0.11, 0.47] *
Holdout	SVR	0.08 [0.04, 0.13] *	−0.02 [−0.05, 0.01]	0.03 [0.00, 0.06] *	−0.10 [−0.39, 0.10]	0.26 [0.12, 0.49] *
Holdout	MLP	0.09 [0.04, 0.15] *	−0.03 [−0.07, 0.00]	0.03 [−0.00, 0.06]	−0.18 [−0.49, 0.04]	0.30 [0.14, 0.53] *
Holdout	Gradient boosting	0.08 [0.04, 0.13] *	−0.03 [−0.06, −0.01] †	0.03 [−0.00, 0.06]	−0.18 [−0.45, −0.01] †	0.26 [0.11, 0.48] *
Holdout	XGBoost	0.09 [0.04, 0.15] *	−0.03 [−0.06, −0.01] †	0.03 [0.00, 0.06] *	−0.18 [−0.45, −0.01] †	0.30 [0.14, 0.54] *
(**b**)						
**Split**	**ML model**	**ΔSens.**	**ΔSpec.**	**ΔBA**	**Δlog10LR+**	**Δlog10LR_below**
OOF	Linear regression	−0.86 [−0.92, −0.80] †	0.04 [0.03, 0.06] ‡	−0.41 [−0.44, −0.38] †	NE	−0.83 [−1.06, −0.67] †
OOF	Decision tree	0.00 [0.00, 0.00]	−0.00 [−0.00, 0.00]	−0.00 [−0.00, 0.00]	−0.01 [−0.04, 0.00]	−0.00 [−0.00, 0.00]
OOF	Random forest	−0.86 [−0.92, −0.79] †	0.04 [0.03, 0.06] ‡	−0.41 [−0.44, −0.38] †	NE	−0.83 [−1.07, −0.66] †
OOF	SVR	−0.01 [−0.03, 0.00]	0.00 [0.00, 0.00]	−0.00 [−0.01, 0.00]	0.01 [−0.01, 0.03]	−0.03 [−0.09, 0.00]
OOF	MLP	−0.27 [−0.35, −0.19] †	0.02 [0.01, 0.02] *	−0.13 [−0.17, −0.09] †	0.04 [−0.07, 0.18]	−0.45 [−0.66, −0.31] †
OOF	Gradient boosting	−0.01 [−0.03, 0.00]	0.00 [0.00, 0.00]	−0.00 [−0.01, 0.00]	0.01 [−0.01, 0.03]	−0.03 [−0.09, 0.00]
OOF	XGBoost	−0.01 [−0.03, 0.00]	0.00 [0.00, 0.00]	−0.00 [−0.01, 0.00]	0.01 [−0.01, 0.03]	−0.03 [−0.09, 0.00]
Holdout	Linear regression	−0.79 [−0.93, −0.65] †	0.04 [0.02, 0.07] ‡	−0.37 [−0.44, −0.29] †	NE	−0.64 [−1.05, −0.41] †
Holdout	Decision tree	0.00 [0.00, 0.00]	0.00 [0.00, 0.00]	0.00 [0.00, 0.00]	0.00 [0.00, 0.00]	0.00 [0.00, 0.00]
Holdout	Random forest	−0.79 [−0.93, −0.62] †	0.04 [0.02, 0.07] ‡	−0.37 [−0.44, −0.29] †	NE	−0.64 [−1.05, −0.39] †
Holdout	SVR	0.00 [0.00, 0.00]	0.00 [0.00, 0.01]	0.00 [0.00, 0.01]	0.04 [0.00, 0.15]	0.00 [0.00, 0.01]
Holdout	MLP	0.00 [0.00, 0.00]	0.00 [0.00, 0.01]	0.00 [0.00, 0.01]	0.04 [0.00, 0.16]	0.00 [0.00, 0.01]
Holdout	Gradient boosting	0.00 [0.00, 0.00]	0.00 [0.00, 0.01]	0.00 [0.00, 0.01]	0.04 [0.00, 0.15]	0.00 [0.00, 0.01]
Holdout	XGBoost	0.00 [0.00, 0.00]	0.00 [0.00, 0.01]	0.00 [0.00, 0.01]	0.04 [0.00, 0.15]	0.00 [0.00, 0.01]
(**c**)						
**Split**	**ML model**	**ΔSens.**	**ΔSpec.**	**ΔBA**	**Δlog10LR+**	**Δlog10LR_below**
OOF	Linear regression	0.01 [−0.01, 0.03]	0.02 [0.00, 0.04] *	0.01 [0.00, 0.03] *	0.08 [0.01, 0.15] *	0.07 [−0.05, 0.21]
OOF	Decision tree	−0.01 [−0.04, 0.01]	0.04 [0.02, 0.06] *	0.01 [−0.00, 0.03]	0.14 [0.06, 0.22] *	−0.06 [−0.18, 0.06]
OOF	Random forest	−0.01 [−0.03, 0.01]	0.04 [0.02, 0.05] *	0.01 [0.00, 0.03] *	0.14 [0.07, 0.22] *	−0.02 [−0.14, 0.10]
OOF	SVR	−0.01 [−0.03, 0.01]	0.04 [0.02, 0.05] *	0.01 [−0.00, 0.03]	0.14 [0.06, 0.23] *	−0.03 [−0.15, 0.08]
OOF	Neural network	−0.05 [−0.07, −0.02] †	0.04 [0.02, 0.06] *	−0.00 [−0.02, 0.01]	0.15 [0.06, 0.25] *	−0.19 [−0.33, −0.07] †
OOF	Gradient boosting	−0.01 [−0.03, 0.01]	0.04 [0.02, 0.05] *	0.01 [−0.00, 0.03]	0.14 [0.06, 0.22] *	−0.05 [−0.17, 0.06]
OOF	XGBoost	−0.01 [−0.03, 0.01]	0.03 [0.01, 0.05] *	0.01 [−0.00, 0.03]	0.13 [0.06, 0.22] *	−0.03 [−0.15, 0.08]
Holdout	Linear regression	−0.01 [−0.04, 0.03]	0.04 [0.00, 0.08]	0.01 [−0.01, 0.04]	0.15 [0.00, 0.34]	−0.03 [−0.29, 0.21]
Holdout	Decision tree	−0.03 [−0.07, 0.02]	0.04 [0.00, 0.08]	0.01 [−0.02, 0.04]	0.14 [−0.02, 0.35]	−0.11 [−0.41, 0.12]
Holdout	Random forest	−0.03 [−0.07, 0.01]	0.04 [0.01, 0.08] *	0.01 [−0.02, 0.04]	0.17 [0.04, 0.36] *	−0.11 [−0.36, 0.08]
Holdout	SVR	−0.03 [−0.07, 0.01]	0.06 [0.03, 0.10] *	0.02 [−0.01, 0.05]	0.28 [0.12, 0.52] *	−0.10 [−0.36, 0.09]
Holdout	Neural network	−0.02 [−0.06, 0.02]	0.05 [0.01, 0.10] *	0.02 [−0.01, 0.05]	0.21 [0.03, 0.44] *	−0.07 [−0.35, 0.16]
Holdout	Gradient boosting	−0.03 [−0.07, 0.02]	0.05 [0.01, 0.09] *	0.01 [−0.02, 0.04]	0.20 [0.05, 0.43] *	−0.11 [−0.39, 0.12]
Holdout	XGBoost	−0.02 [−0.06, 0.02]	0.05 [0.01, 0.09] *	0.02 [−0.01, 0.04]	0.21 [0.05, 0.41] *	−0.07 [−0.35, 0.16]
(**d**)						
**Split**	**ML model**	**ΔSens.**	**ΔSpec.**	**ΔBA**	**Δlog10LR+**	**Δlog10LR_below**
OOF	Linear regression	−0.95 [−0.98, −0.90] †	0.09 [0.07, 0.11] ‡	−0.43 [−0.45, −0.41] †	NE	−1.21 [−1.63, −0.96] †
OOF	Decision tree	−0.09 [−0.14, −0.04] †	0.05 [0.04, 0.06] *	−0.02 [−0.05, 0.01]	0.28 [0.19, 0.39] *	−0.38 [−0.77, −0.17] †
OOF	Random forest	−0.95 [−0.98, −0.90] †	0.09 [0.07, 0.11] ‡	−0.43 [−0.45, −0.40] †	NE	−1.21 [−1.63, −0.96] †
OOF	SVR	−0.10 [−0.16, −0.05] †	0.05 [0.04, 0.06] *	−0.02 [−0.05, 0.00]	0.30 [0.20, 0.41] *	−0.41 [−0.78, −0.20] †
OOF	Neural network	−0.36 [−0.44, −0.27] †	0.07 [0.05, 0.08] *	−0.15 [−0.19, −0.10] †	0.33 [0.19, 0.50] *	−0.83 [−1.27, −0.59] †
OOF	Gradient boosting	−0.10 [−0.16, −0.04] †	0.05 [0.04, 0.06] *	−0.02 [−0.05, 0.00]	0.30 [0.20, 0.41] *	−0.41 [−0.80, −0.19] †
OOF	XGBoost	−0.10 [−0.15, −0.04] †	0.05 [0.04, 0.06] *	−0.02 [−0.05, 0.00]	0.30 [0.21, 0.41] *	−0.41 [−0.78, −0.19] †
Holdout	Linear regression	−0.83 [−0.97, −0.69] †	0.09 [0.06, 0.13] ‡	−0.37 [−0.43, −0.29] †	NE	−0.69 [−1.24, −0.44] †
Holdout	Decision tree	−0.03 [−0.10, 0.00]	0.05 [0.02, 0.08] *	0.01 [−0.03, 0.03]	0.29 [0.13, 0.53] *	−0.05 [−0.25, 0.03]
Holdout	Random forest	−0.83 [−0.97, −0.69] †	0.09 [0.06, 0.13] ‡	−0.37 [−0.43, −0.29] †	NE	−0.69 [−1.24, −0.44] †
Holdout	SVR	−0.03 [−0.10, 0.00]	0.05 [0.03, 0.08] *	0.01 [−0.03, 0.04]	0.33 [0.16, 0.57] *	−0.05 [−0.25, 0.03]
Holdout	Neural network	−0.03 [−0.10, 0.00]	0.05 [0.03, 0.08] *	0.01 [−0.03, 0.04]	0.33 [0.16, 0.58] *	−0.05 [−0.25, 0.03]
Holdout	Gradient boosting	−0.03 [−0.10, 0.00]	0.05 [0.03, 0.08] *	0.01 [−0.03, 0.04]	0.33 [0.16, 0.59] *	−0.05 [−0.25, 0.03]
Holdout	XGBoost	−0.03 [−0.10, 0.00]	0.05 [0.02, 0.08] *	0.01 [−0.03, 0.04]	0.33 [0.16, 0.59] *	−0.05 [−0.25, 0.03]

(**a**) Values are Δ = ML model − reference method with 95% bootstrap confidence intervals. Positive values favor the ML model and negative values favor the reference method, because higher Sens., Spec., BA, log10LR+, and log10LR_below indicate better performance. * Significant difference favoring ML; † significant difference favoring the reference method. LR_below = 1/LR−. (**b**) Values are Δ = ML model − reference method with 95% bootstrap confidence intervals. Positive values favor the ML model and negative values favor the reference method, because higher Sens., Spec., BA, log10LR+, and log10LR_below indicate better performance. * Significant difference favoring ML; † significant difference favoring the reference method. LR_below = 1/LR−. NE: not estimable because the model produced no positive predictions at the 15-year threshold. ‡ No positive predictions at the 15-year threshold; high specificity should not be interpreted as superior threshold performance. (**c**) Values are Δ = ML model − reference method with 95% bootstrap confidence intervals. Positive values favor the ML model and negative values favor the reference method, because higher Sens., Spec., BA, log10LR+, and log10LR_below indicate better performance. * Significant difference favoring ML; † significant difference favoring the reference method. LR_below = 1/LR−. (**d**) Values are Δ = ML model − reference method with 95% bootstrap confidence intervals. Positive values favor the ML model and negative values favor the reference method, because higher Sens., Spec., BA, log10LR+, and log10LR_below indicate better performance. * Significant difference favoring ML; † significant difference favoring the reference method. LR_below = 1/LR−. NE: not estimable because the model produced no positive predictions at the 15-year threshold. ‡ No positive predictions at the 15-year threshold; high specificity should not be interpreted as superior threshold performance.

## Data Availability

The de-identified tabular data supporting the findings of this study are available from the corresponding author upon reasonable request. The original panoramic radiographs are not publicly available because they contain sensitive clinical information and are subject to patient privacy, ethical approval, and institutional data-protection restrictions.

## Supplementary Materials

The following supporting information can be downloaded at: https://www.mdpi.com/article/10.3390/diagnostics16172690/s1, Supplementary Table S1: Computational environment, model-development settings, preprocessing, hyperparameter search spaces, and selected hyperparameters. Supplementary File S2: Extended Agreement, Calibration, and Threshold-Classification Analysis for Dental Age Estimation: Demirjian, Willems, and Machine Learning Regression Models.
