# Supplementary material for: Does Lower Regression Error Mean Stronger Forensic Evidence? Machine Learning Regression Versus Demirjian and Willems Methods for Dental Age Estimation at 12- and 15-Year Legal Thresholds
Source: Diagnostics (Basel). 2026 Aug 23;16(17):2690. doi: 10.3390/diagnostics16172690 (PMC13565120; doi:10.3390/diagnostics16172690)
Supplement: Supplementary file 1 [file diagnostics-16-02690-s001.zip › SupplementaryFileS2.pdf]

Supplementary File S2

Extended Agreement, Calibration, and Threshold-Classification Analysis for Dental Age Estimation: Demirjian, Willems, and Machine Learning Regression Models

S2.0 Purpose and scope

This supplementary file extends the pooled error metrics (ME, MAE, RMSE, R^2) reported in Table 1 of the main manuscript with additional analyses of agreement, calibration, age-specific prediction error, and threshold-classification behavior. Three complementary questions are addressed: (i) how closely the compared methods agree with chronological age across the observed age range; (ii) whether prediction error and residual dispersion vary across chronological-age strata; and (iii) how threshold classifications behave across age strata surrounding the 12- and 15-year cut-offs.

Analyses are reported separately for the training subset (n = 1107) and the Internal Holdout set (n = 277). For the machine-learning models, training-subset results are based on out-of-fold (OOF) predictions generated by five-fold cross-validation; the Willems and Demirjian values are direct method estimates calculated for the same training-subset individuals. The Internal Holdout set was not used for model fitting, hyperparameter tuning, or model selection. Chronological-age (CA) strata are 1-year-wide and were computed from the observed age range; strata with no observations are omitted from figures and tables.

Willems and Demirjian estimates are included alongside the seven machine learning regressors (LR = linear regression, DT = decision tree, RF = random forest, SVR = support vector regression, MLP = multilayer perceptron, GB = gradient boosting, XGB = extreme gradient boosting) throughout, so that the classical staging methods can be judged on the same terms.

Contents

- S2.1 — Age-stratified mean error and mean absolute error
- S2.2 — Bland–Altman agreement analysis
- S2.3 — Global calibration relationship (linear and non-linear diagnostic)
- S2.4 — Age-specific descriptive error dispersion
- S2.5 — Threshold-classification profiles at the 12- and 15-year legal thresholds
- S2.6 — Summary tables
- S2.7 — Glossary: how to read these figures

S2.1 Age-stratified mean error and mean absolute error

Figures S2-1 and S2-2 show, for each 1-year chronological-age stratum, the mean signed error (ME = predicted age − CA, above) and mean absolute error (MAE = |predicted age − CA|, below) for every method, plotted as the bootstrap-mean point estimate with a 95% confidence-interval whisker (2000 resamples, percentile method).

The age-stratified results show that pooled error metrics can conceal systematic changes in prediction error across the observed age range. Several methods show a tendency toward greater underestimation in the older strata, particularly near the upper end of the study range. These age-dependent patterns are consistent with the regression-toward-the-mean behavior discussed in the main manuscript and provide a direct view of the prediction errors underlying the threshold-classification results.

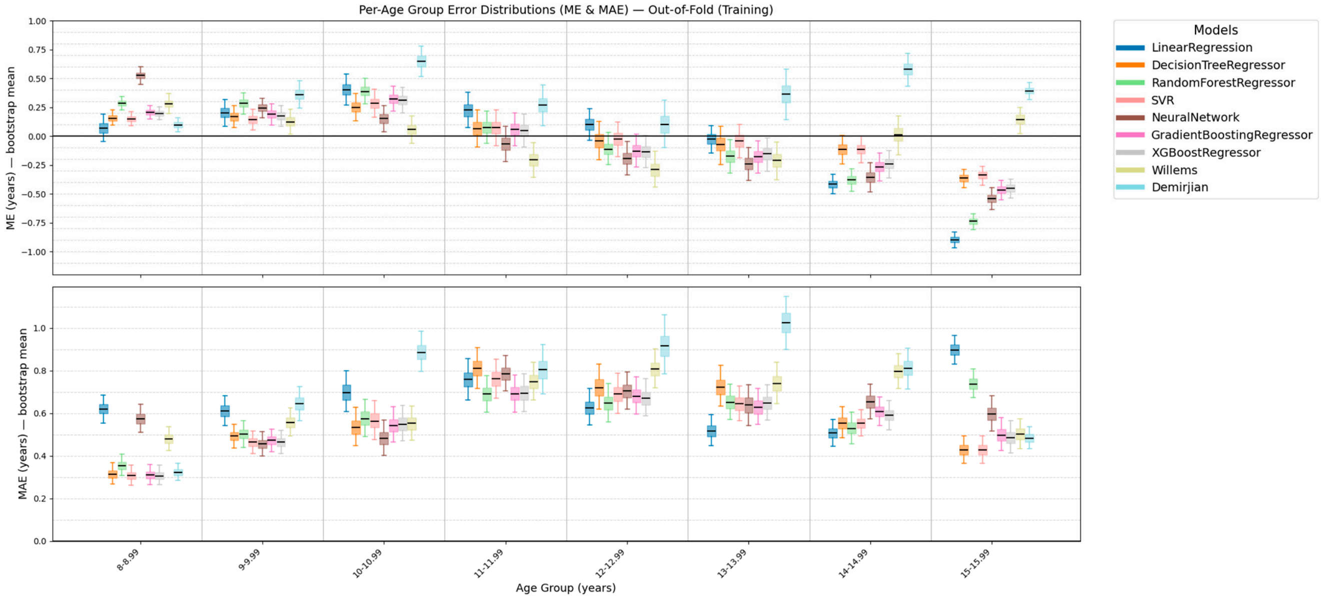

Figure S2-1. Mean error (ME, above) and mean absolute error (MAE, below) by chronological-age stratum, Training subset (OOF predictions for ML models). Points are bootstrap-mean estimates with 95% CI whiskers.

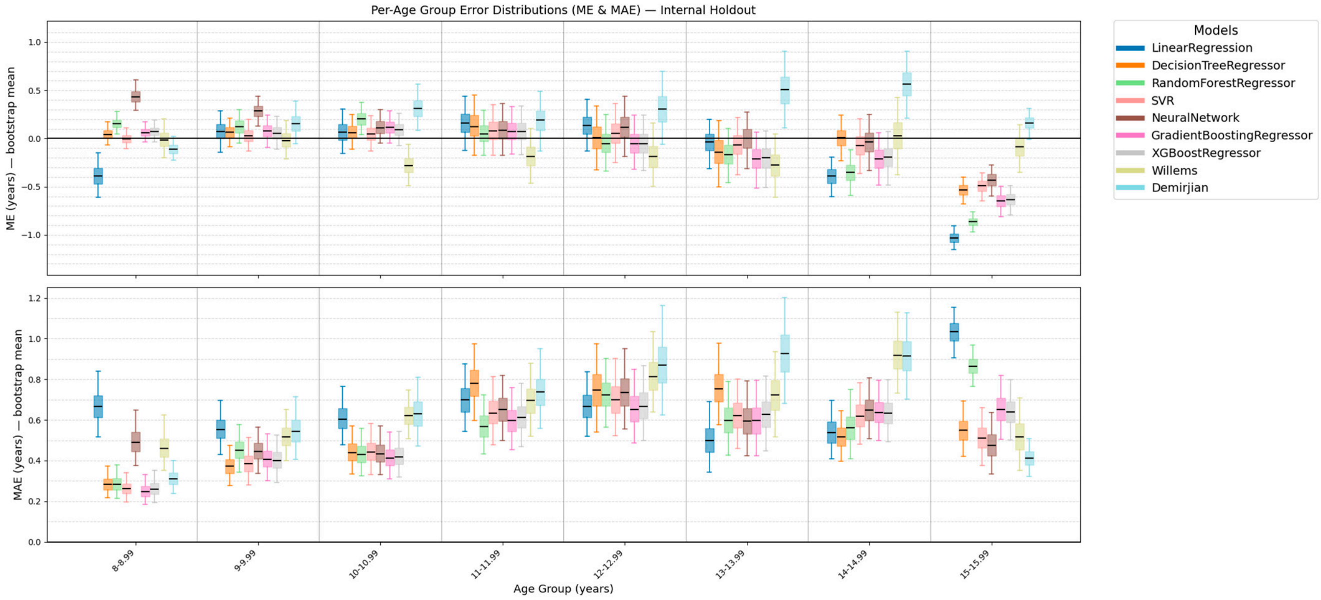

Figure S2-2. Mean error (ME, above) and mean absolute error (MAE, below) by chronological-age stratum, Internal Holdout set. Points are bootstrap-mean estimates with 95% CI whiskers.

S2.2 Bland–Altman agreement analysis

For each method, Figures S2-3 (OOF) and S2-4 (Holdout) plot the difference between predicted age and chronological age (y-axis) against the mean of the two (x-axis), following the standard Bland–Altman convention. The solid black line marks the bias (mean difference); the dashed red lines mark the 95% limits of agreement (bias ± 1.96 × SD of the differences); the dotted grey line marks zero difference.

A visible diagonal banding pattern is present for Willems, Demirjian, and the tree-based regressors (DT, RF, GB, XGB). This is an expected property of those methods rather than a plotting artefact: staging systems such as Willems and Demirjian assign a fixed predicted age to each developmental stage, and tree-based models predict the average age of the training samples that fall in the same leaf node — both mechanisms produce predictions drawn from a limited, discrete set of values, which appear as parallel diagonal streaks when plotted against a continuous chronological age.

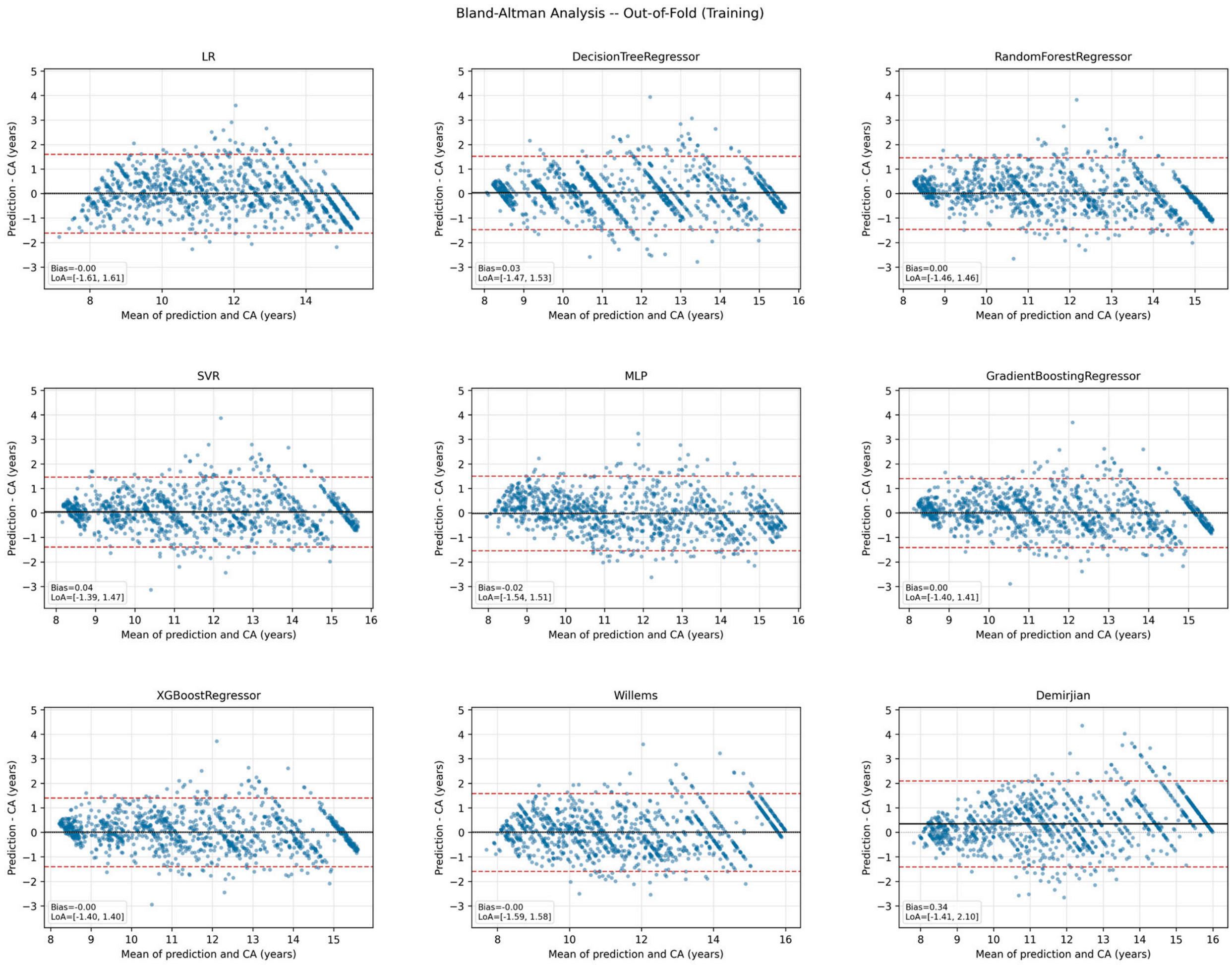

Figure S2-3. Bland–Altman plots, Training subset (OOF predictions for ML models). Bias and 95% limits of agreement (LoA) are annotated on each panel; numerical values are also reported in Table S2.2.

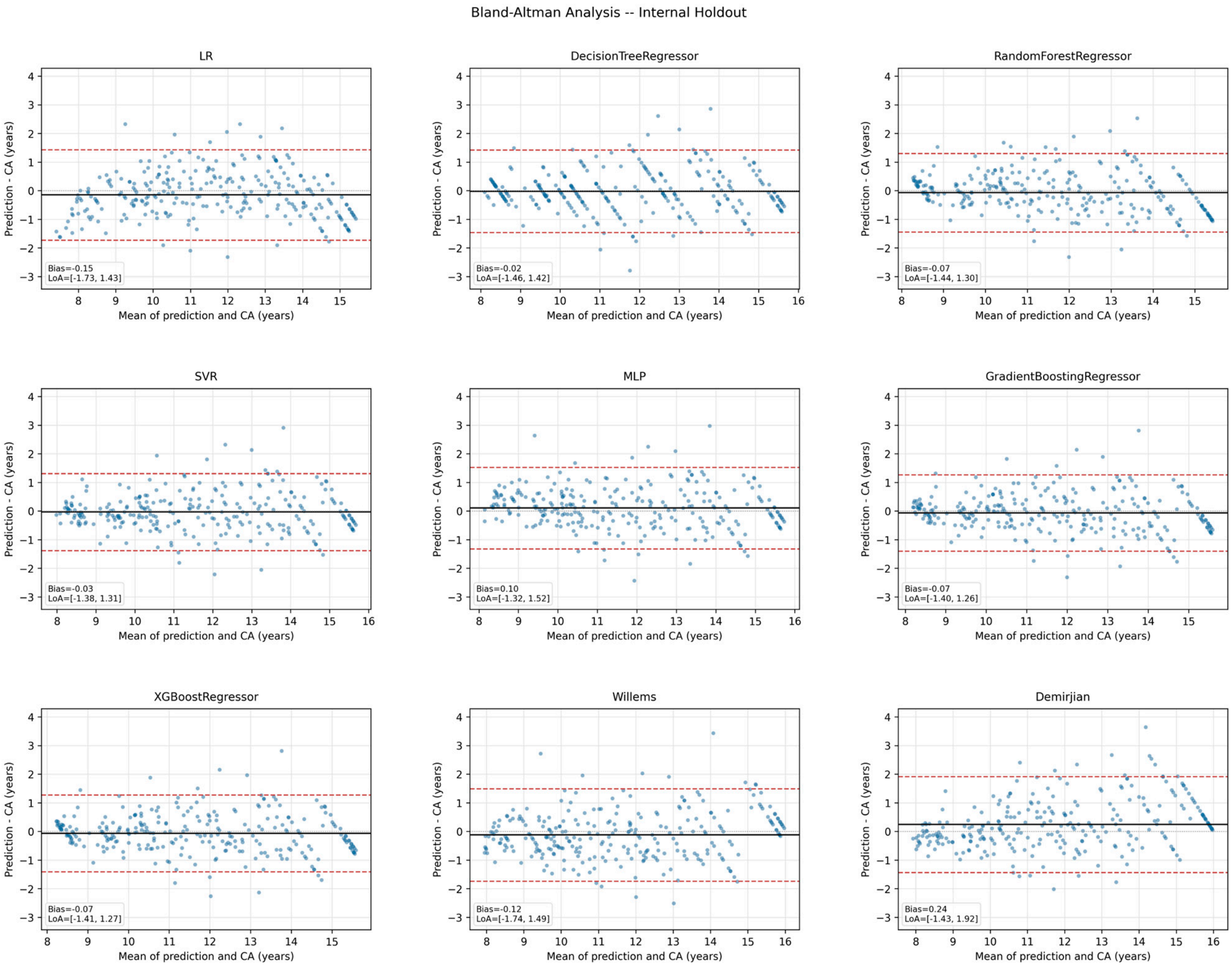

Figure S2-4. Bland–Altman plots, Internal Holdout set.

S2.3 Global calibration relationship (linear and non-linear diagnostic)

Figures S2-5 and S2-6 plot predicted age against chronological age for every method, together with: a linear ordinary-least-squares (OLS) fit (solid black line) with its 95% confidence band for the mean relationship (shaded blue) and its 95% prediction interval (dashed red lines, statsmodels get_prediction().summary_frame()); a locally weighted regression curve (LOWESS, dash-dot green) shown purely as a visual diagnostic for non-linearity; and the identity line (dotted grey, predicted age = CA) as a reference for perfect calibration. All nine panels within a figure share the same x- and y-axis range, so that the shape and position of each method's fit can be compared directly.

The LOWESS curve is not used to compute any interval or statistic reported elsewhere in this file or in the main text — the formally reported 95% CI/PI is always the linear-OLS band. Its purpose is only to make visible any departure from a straight-line relationship, such as the mild flattening of the fitted curve toward the upper end of the age range visible for several methods, consistent with a ceiling effect as dental maturity indicators approach their developmental limit.

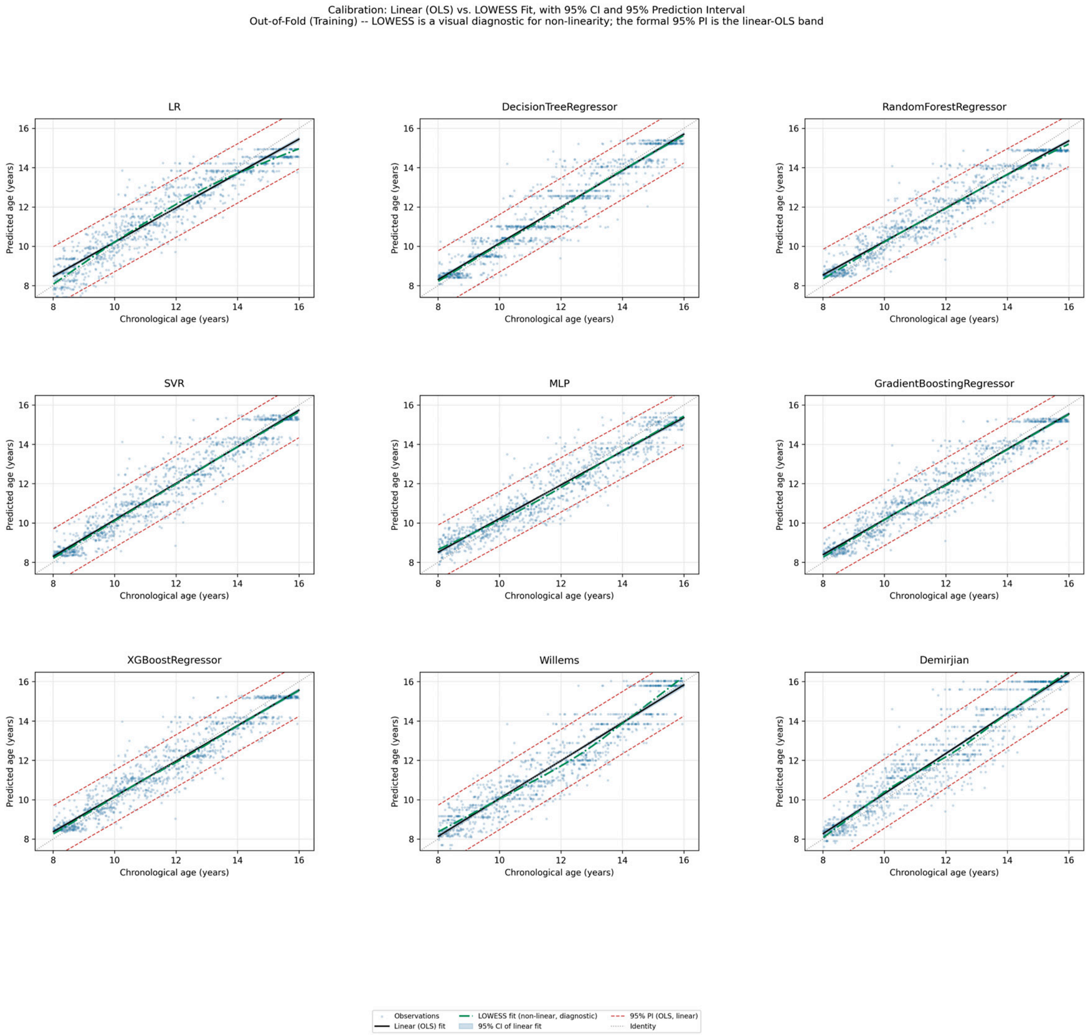

Figure S2-5. Predicted age vs. chronological age with linear (OLS) and LOWESS fits, Training subset (OOF predictions for ML models).

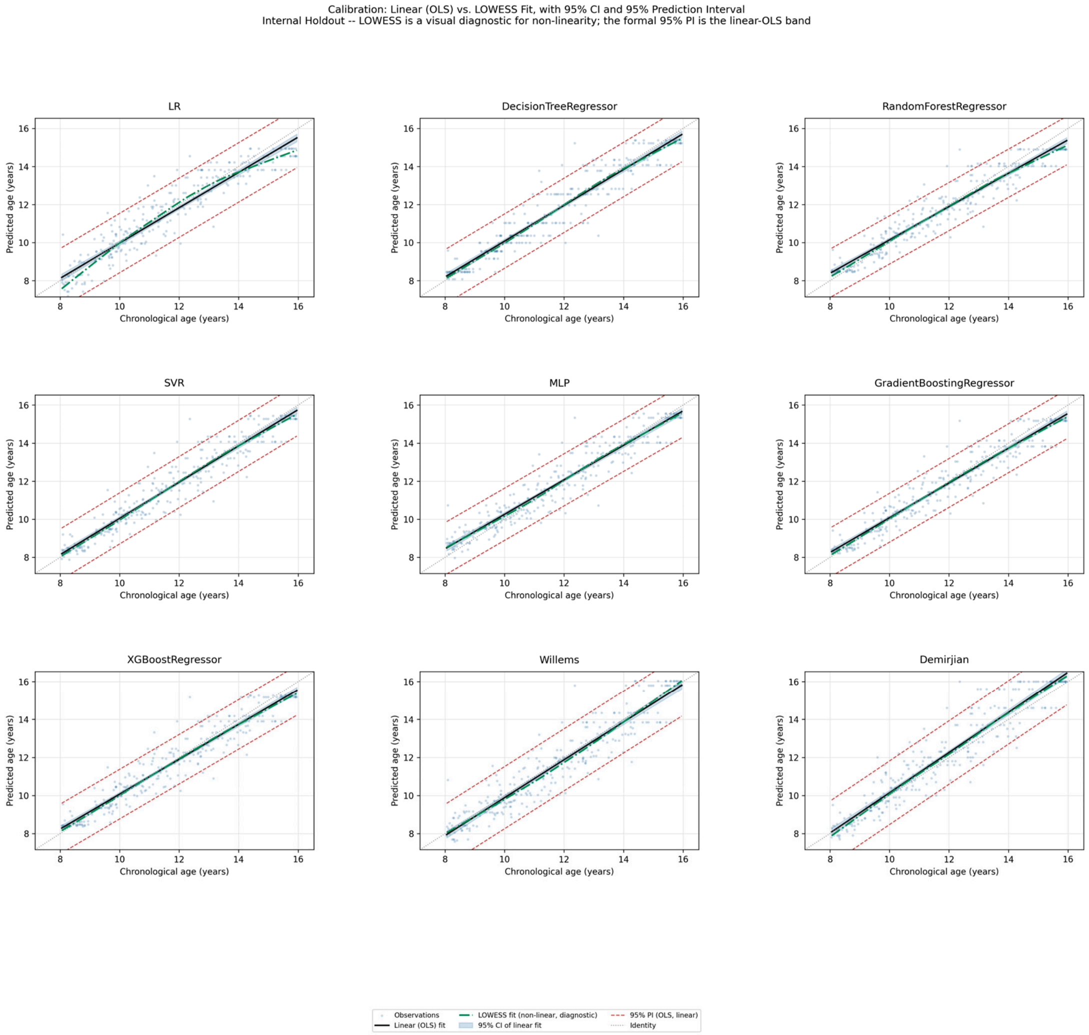

Figure S2-6. Predicted age vs. chronological age with linear (OLS) and LOWESS fits, Internal Holdout set.

S2.4 Age-specific descriptive error dispersion

For each chronological-age stratum, Figures S2-7 and S2-8 show two different quantities on the same axis, and it is important not to conflate them. The narrow black error bar is the bootstrap 95% confidence interval of the mean error (ME) in that stratum — i.e., the uncertainty in the average bias, given the sample size available in that 1-year age band (2000 resamples, percentile method). The wide pink bar is the descriptive residual dispersion, ME ± 1.96 × SD, which summarizes how spread out individual errors are within that stratum.

The wide dispersion interval is descriptive rather than predictive: it summarizes the observed spread of individual residuals within each chronological-age stratum and should not be interpreted as a formally validated prediction interval for a new individual. Its width is estimated from the observations available in that stratum and may therefore be less stable in smaller age groups.

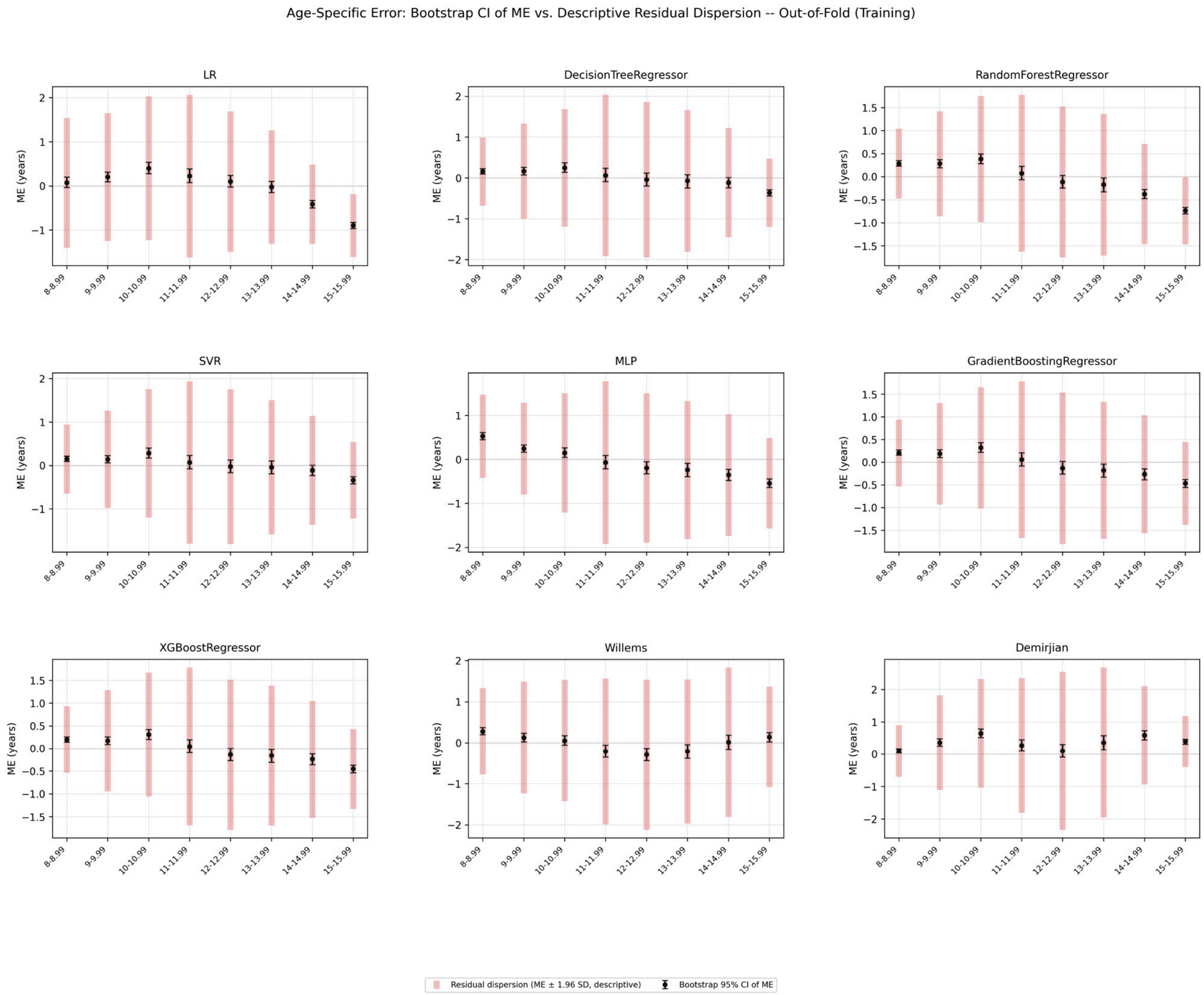

Figure S2-7. Age-specific mean error with bootstrap 95% CI (narrow bar) and descriptive residual dispersion, ME ± 1.96 SD (wide bar), Training subset (OOF predictions for ML models).

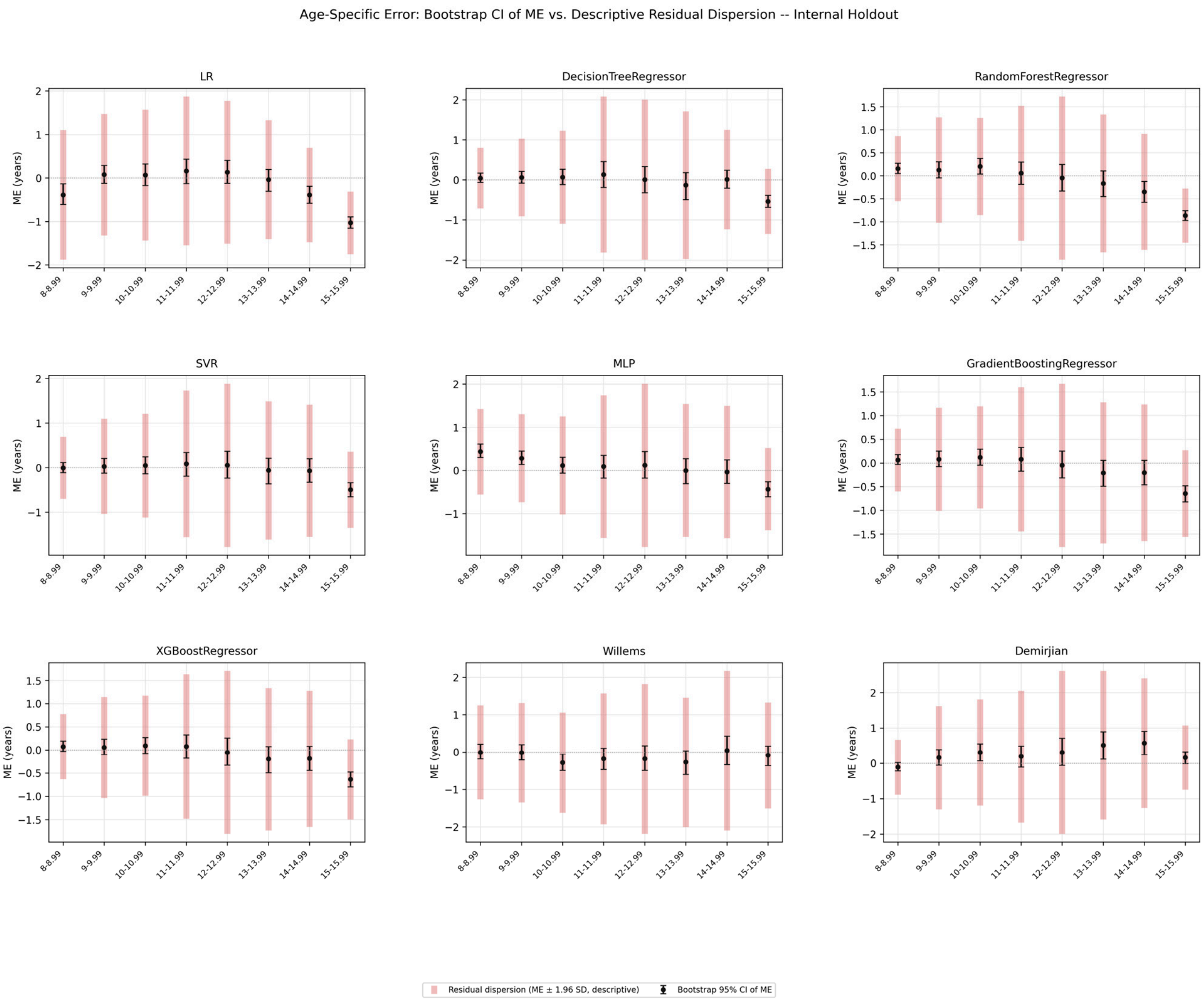

Figure S2-8. Age-specific mean error with bootstrap 95% CI (narrow bar) and descriptive residual dispersion (wide bar), Internal Holdout set.

S2.5 Threshold-classification profiles at the 12- and 15-year legal thresholds

Figures S2-9 to S2-12 give an age-stratified view of the threshold classification that underlies the sensitivity/specificity results in the main text. For each chronological-age stratum and each method, the blue line shows the proportion of individuals predicted to be at or above the legal threshold (12 or 15 years), with an exact (Clopper–Pearson) binomial 95% confidence interval; the black dashed line shows the true proportion at or above threshold in that stratum, based on chronological age (a step function: 0 below the threshold stratum and 1 at and above it).

A method that tracked chronological age perfectly would show its blue curve changing from 0 to 1 at the same stratum as the black dashed reference. Departures from this pattern identify the age strata contributing to false-positive classifications (predicted at or above the threshold despite chronological age below it) and false-negative classifications (predicted below the threshold despite chronological age at or above it). False-negative behavior at the 15-year threshold was most pronounced for LR and RF, which generated no positive classifications in the main threshold analysis, whereas the remaining methods showed varying but substantially higher proportions of positive classifications in the 15.00–15.99-year group.

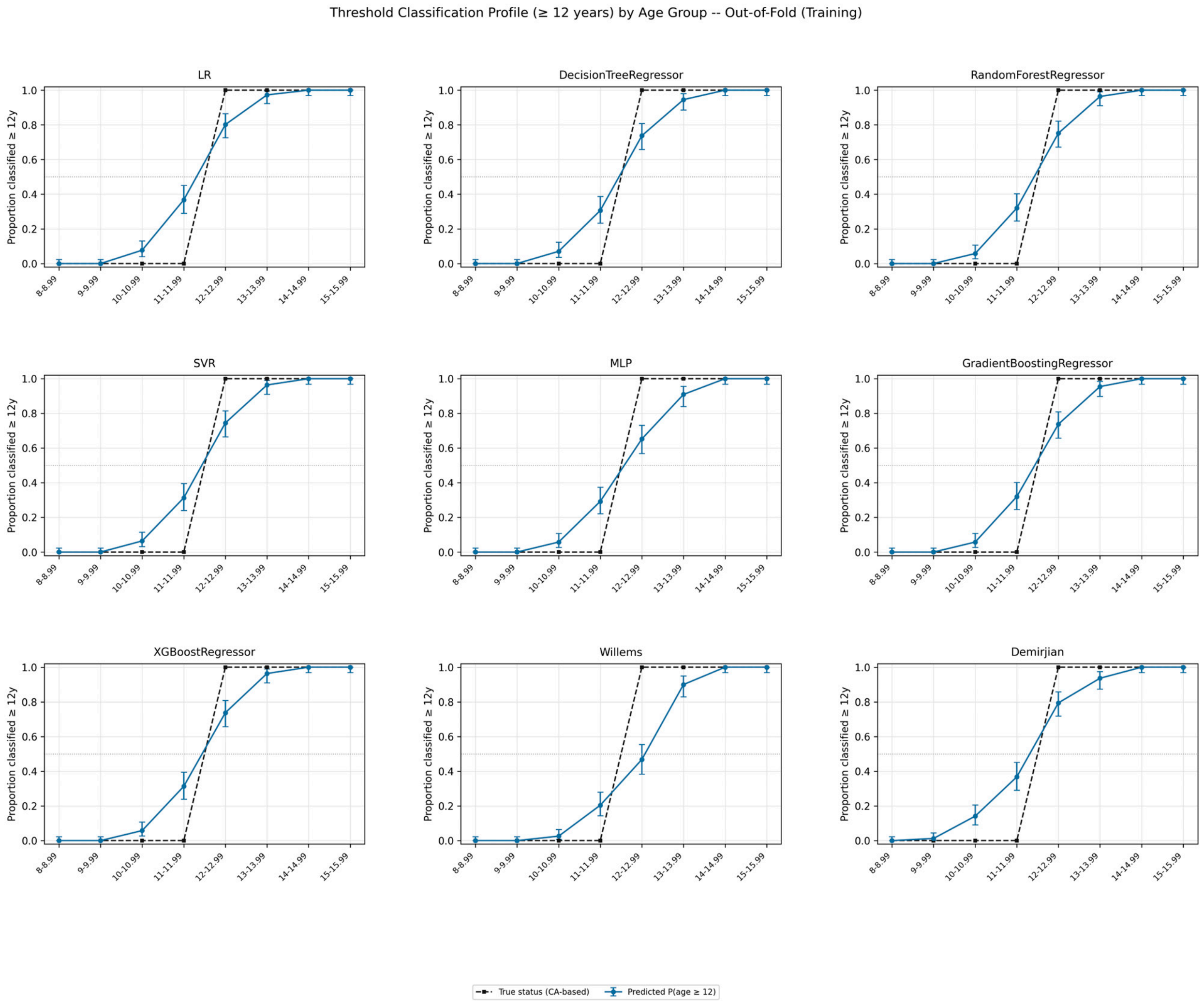

Figure S2-9. Threshold classification profile (≥ 12 years) by chronological-age stratum, Training subset (OOF predictions for ML models).

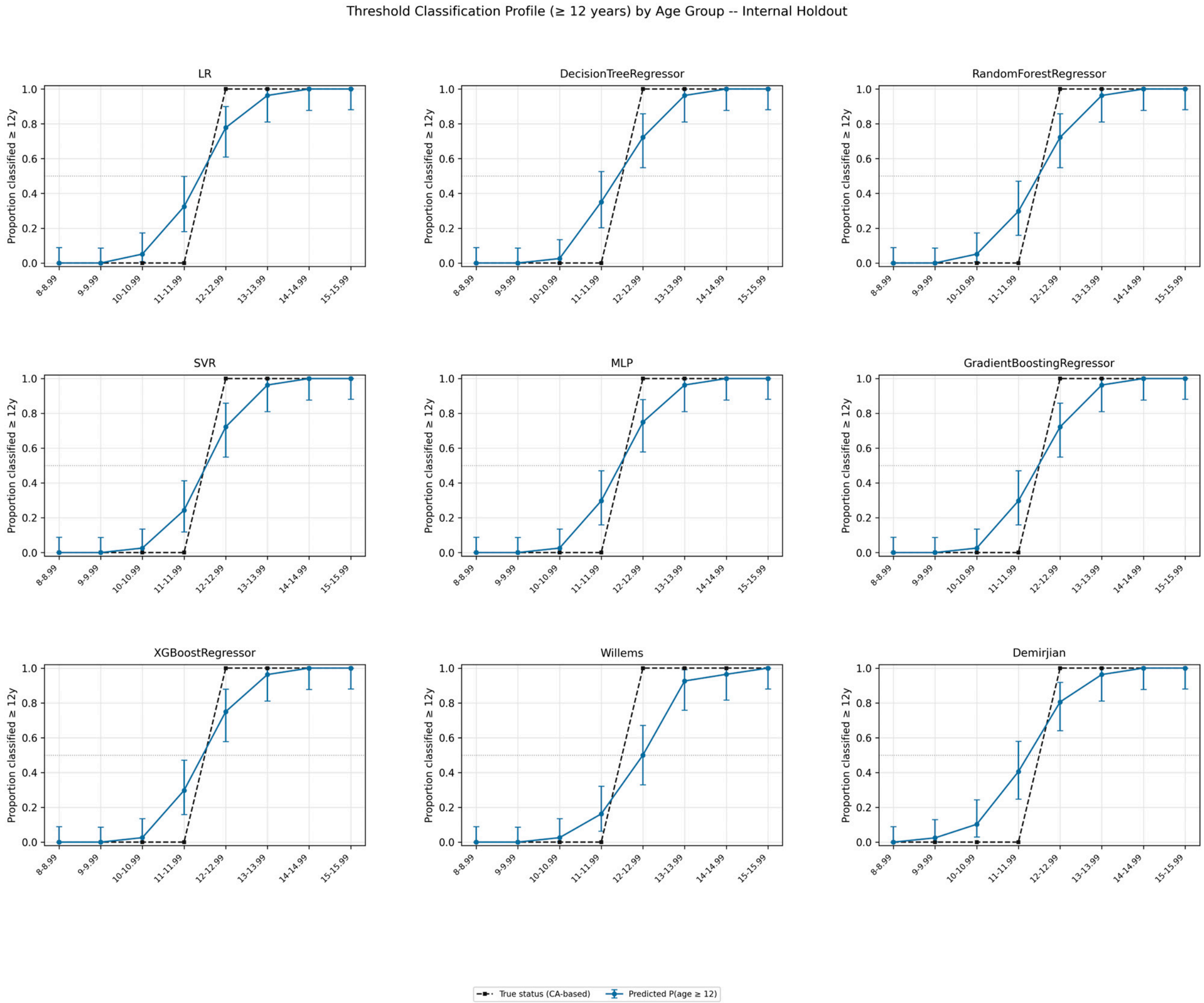

Figure S2-10. Threshold classification profile (≥ 12 years) by chronological-age stratum, Internal Holdout set.

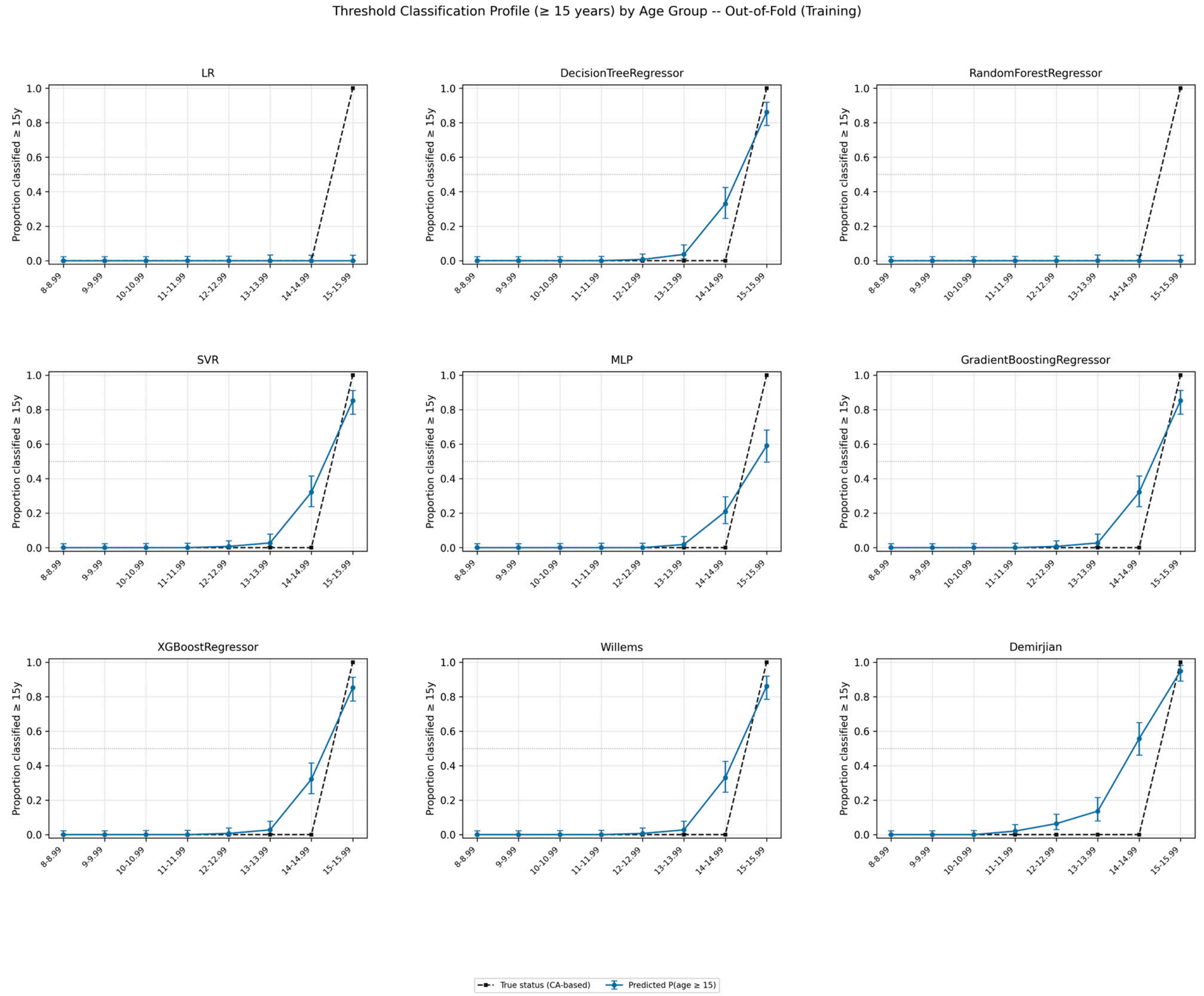

Figure S2-11. Threshold classification profile (≥ 15 years) by chronological-age stratum, Training subset (OOF predictions for ML models).

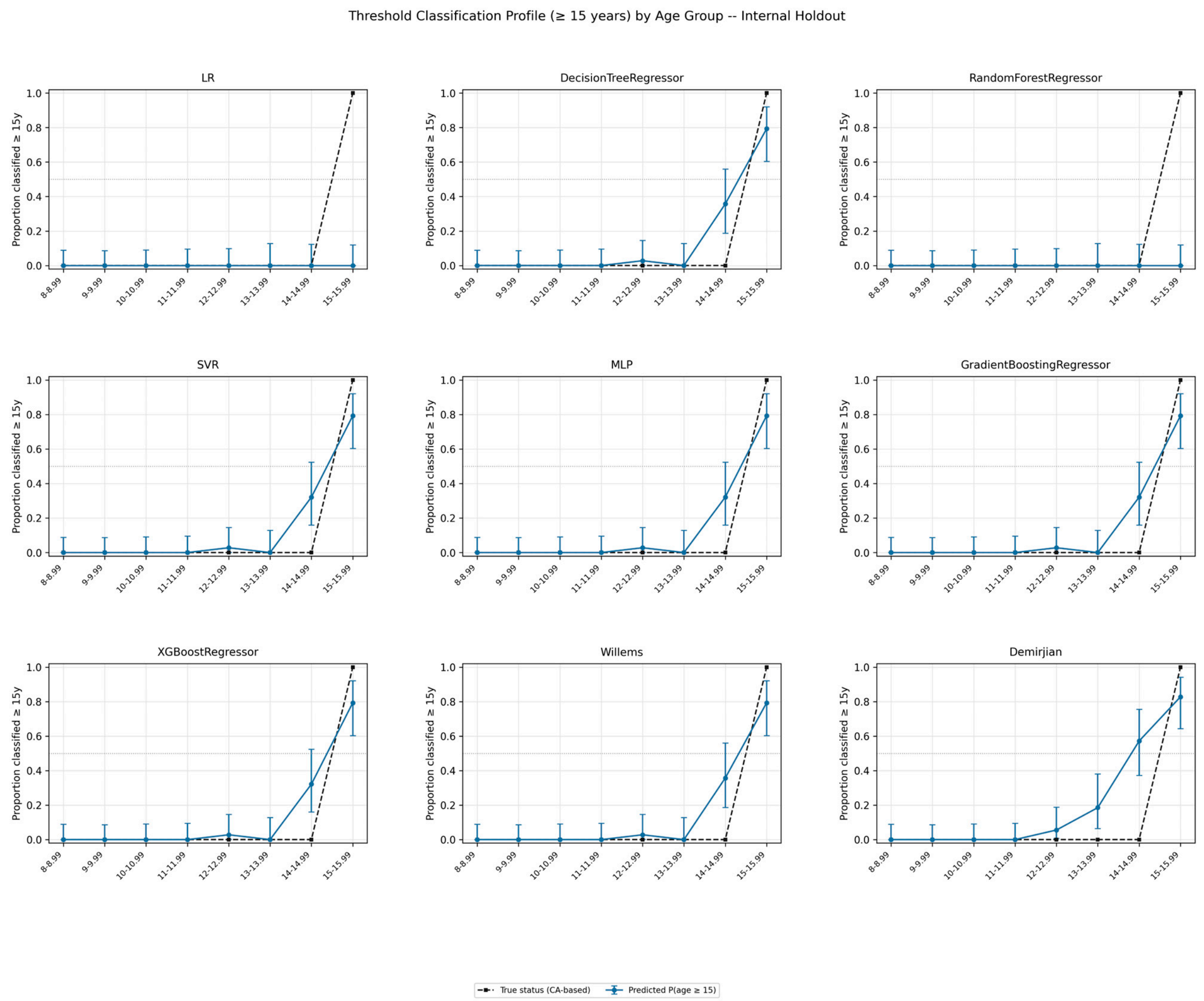

Figure S2-12. Threshold classification profile (≥ 15 years) by chronological-age stratum, Internal Holdout set.

S2.6 Summary tables

Note on split labels: "OOF" denotes out-of-fold predictions for the ML models; Willems and Demirjian values in the corresponding rows are direct estimates calculated for the same training-subset individuals.

Table S2.1. Overall error statistics by model and split

ME and MAE are reported with bootstrap 95% confidence intervals (2000 resamples, percentile method).

Split	Model	n	ME [95% CI] (y)	MAE [95% CI] (y)	RMSE (y)	R^2
OOF	LR	1107	-0.00 [-0.05, 0.05]	0.66 [0.63, 0.69]	0.82	0.872
OOF	DT	1107	0.03 [-0.01, 0.07]	0.57 [0.54, 0.60]	0.76	0.888
OOF	RF	1107	0.00 [-0.04, 0.04]	0.58 [0.55, 0.61]	0.74	0.894
OOF	SVR	1107	0.04 [-0.01, 0.08]	0.55 [0.52, 0.58]	0.73	0.899
OOF	MLP	1107	-0.02 [-0.06, 0.03]	0.61 [0.58, 0.64]	0.78	0.885
OOF	GB	1107	0.00 [-0.04, 0.04]	0.55 [0.52, 0.57]	0.72	0.902
OOF	XGB	1107	-0.00 [-0.04, 0.04]	0.54 [0.52, 0.57]	0.71	0.902
OOF	Willems	1107	-0.00 [-0.05, 0.05]	0.64 [0.61, 0.67]	0.81	0.875
OOF	Demirjian	1107	0.34 [0.29, 0.40]	0.73 [0.69, 0.76]	0.96	0.824
Holdout	LR	277	-0.15 [-0.24, -0.05]	0.66 [0.60, 0.72]	0.82	0.872
Holdout	DT	277	-0.02 [-0.11, 0.06]	0.55 [0.49, 0.61]	0.73	0.897
Holdout	RF	277	-0.07 [-0.15, 0.02]	0.55 [0.50, 0.60]	0.70	0.905
Holdout	SVR	277	-0.03 [-0.11, 0.05]	0.51 [0.46, 0.57]	0.69	0.909
Holdout	MLP	277	0.10 [0.02, 0.19]	0.56 [0.50, 0.62]	0.73	0.897
Holdout	GB	277	-0.07 [-0.14, 0.02]	0.51 [0.46, 0.57]	0.68	0.911
Holdout	XGB	277	-0.07 [-0.15, 0.02]	0.52 [0.47, 0.57]	0.69	0.909
Holdout	Willems	277	-0.12 [-0.21, -0.02]	0.65 [0.59, 0.71]	0.83	0.867
Holdout	Demirjian	277	0.24 [0.14, 0.34]	0.65 [0.59, 0.72]	0.89	0.849

Table S2.2. Bland–Altman summary by model and split

Bias = mean(predicted age − CA); 95% LoA = bias ± 1.96 × SD.

Split	Model	n	Bias (y)	SD (y)	95% LoA (y)
OOF	LR	1107	-0.00	0.82	[-1.61, 1.61]
OOF	DT	1107	0.03	0.76	[-1.47, 1.53]
OOF	RF	1107	0.00	0.75	[-1.46, 1.46]
OOF	SVR	1107	0.04	0.73	[-1.39, 1.47]
OOF	MLP	1107	-0.02	0.78	[-1.54, 1.51]
OOF	GB	1107	0.00	0.72	[-1.40, 1.41]
OOF	XGB	1107	-0.00	0.71	[-1.40, 1.40]
OOF	Willems	1107	-0.00	0.81	[-1.59, 1.58]
OOF	Demirjian	1107	0.34	0.90	[-1.41, 2.10]
Holdout	LR	277	-0.15	0.81	[-1.73, 1.43]
Holdout	DT	277	-0.02	0.74	[-1.46, 1.42]
Holdout	RF	277	-0.07	0.70	[-1.44, 1.30]
Holdout	SVR	277	-0.03	0.69	[-1.38, 1.31]
Holdout	MLP	277	0.10	0.73	[-1.32, 1.52]
Holdout	GB	277	-0.07	0.68	[-1.40, 1.26]
Holdout	XGB	277	-0.07	0.69	[-1.41, 1.27]
Holdout	Willems	277	-0.12	0.82	[-1.74, 1.49]

Split	Model	n	Bias (y)	SD (y)	95% LoA (y)
Holdout	Demirjian	277	0.24	0.85	[-1.43, 1.92]

Full numerical data underlying the supplementary analyses, including age-specific error statistics, Bland–Altman summaries, calibration results, and threshold-classification-by-age data, are provided in the accompanying workbook SupplementaryFileS2_summary.xlsx.

S2.7 Glossary: how to read these figures

Bland–Altman plot

A standard method-agreement plot: each point is one individual, plotted as (mean of the two measures, difference between the two measures). It answers “how much do predicted age and chronological age disagree, and does the disagreement change with age?” rather than “how correlated are they?”, which is why it is preferred over a simple scatter/correlation plot for agreement questions.

Bias and limits of agreement (LoA)

Bias is the average signed error. The 95% limits of agreement describe the range within which about 95% of individual differences fall, under a normal-distribution assumption — they are a description of the current sample's spread, not a validated prediction interval for a new individual.

OLS fit, confidence band, and prediction interval

The OLS (ordinary least squares) line is the best-fitting straight line through predicted age vs. chronological age. Its confidence band describes uncertainty about where that average line itself lies; its prediction interval (always wider) describes the range within which a new individual observation is expected to fall. Both assume the linear model is correctly specified and errors are approximately normally distributed with constant variance.

LOWESS curve

A non-parametric, locally weighted smoothing curve that makes no straight-line assumption. Used here only as a visual check on whether the OLS line's linearity assumption is reasonable — it is not used to compute any reported interval.

Age-specific residual dispersion

A descriptive within-age-stratum interval defined as mean prediction error ± 1.96 residual standard deviations. It summarizes the spread of observed individual errors in that stratum and is not a validated prediction interval for a new individual.

Clopper–Pearson (exact binomial) confidence interval

An exact confidence interval for a proportion (here, the proportion of individuals classified at or above a legal age threshold), which does not rely on a large-sample normal approximation and therefore remains valid even for small strata.

Age-mimicry / regression to the mean

The tendency of a model trained to minimize average error to under-predict the oldest individuals and over-predict the youngest individuals in its training range, because compressing predictions toward the sample mean lowers the average numerical error even though it systematically biases the extremes. This is the mechanism underlying the negative ME seen at the 15-year stratum throughout this file.
