# Supplementary material for: Does Lower Regression Error Mean Stronger Forensic Evidence? Machine Learning Regression Versus Demirjian and Willems Methods for Dental Age Estimation at 12- and 15-Year Legal Thresholds
Source: Diagnostics (Basel). 2026 Aug 23;16(17):2690. doi: 10.3390/diagnostics16172690 (PMC13565120; doi:10.3390/diagnostics16172690)
Supplement: Supplementary file 1 [file diagnostics-16-02690-s001.zip › Supplementary_Table_S1.pdf]

Supplementary Table S1. Computational environment, model-development settings, preprocessing, hyperparameter search spaces, and selected hyperparameters

A. Computational environment

Software/environment	Version/setting
**Execution environment**	Google Colaboratory
**Python**	3.12.13 (CPython)
**NumPy**	2.0.2
**pandas**	2.2.2
**SciPy**	1.16.3
**scikit-learn**	1.6.1
**XGBoost**	3.3.0

B. General model-development and preprocessing settings

Item	Setting
**Input variables**	Sex and Demirjian developmental stages for mandibular teeth 31, 32, 33, 34, 35, 36, and 37.
**Demirjian stage encoding**	Stages A-H were encoded as sequential ordinal integers 1-8 (A=1, B=2, ..., H=8), preserving their ordered developmental progression.
**Sex encoding**	Female=0; male=1.
**Missing data**	No missing values were present in the final analytic dataset; therefore, no imputation was required.
**Feature scaling**	Standard scaling was applied to scale-sensitive models (SVR and MLP). Scaling was not applied to tree-based models (Decision Tree, Random Forest, Gradient Boosting, and XGBoost) or Linear Regression.
**Training/internal holdout split**	80% model-development set and 20% internal holdout test set.
**Split stratification**	Stratification was based on the composite age-group x sex variable.
**Split random seed**	random_state=44.
**Cross-validation**	Five-fold stratified cross-validation within the model-development set, with shuffle=True.
**Cross-validation random seed**	random_state=44.
**Hyperparameter optimization**	GridSearchCV was performed only within the model-development set.
**Model-selection criterion**	Mean squared error (MSE); GridSearchCV refit criterion was neg_mean_squared_error.
**Additional performance metrics**	MAE, RMSE, and R^2 were also calculated.
**Internal holdout use**	The internal holdout test set was not used for hyperparameter tuning or model selection and was accessed only for final internal evaluation.

C. Model-specific hyperparameter search spaces and selected parameters

Model	Scaling	Hyperparameter search space	Selected best parameters	Model random state
**Linear Regression**	No scaling	fit_intercept: [True, False]; positive: [False, True]. Fixed: copy_X=True; n_jobs=-1.	copy_X=True; fit_intercept=True; n_jobs=-1; positive=True	Not applicable
**Decision Tree**	No scaling	criterion: ['squared_error','absolute_error']; max_depth: [3,5,10,None]; min_samples_leaf: [1,2,4]; min_samples_split: [2,4,8]; max_features: [None,'sqrt'].	criterion='absolute_error'; max_depth=5; min_samples_leaf=1; min_samples_split=2	100
**Random Forest**	No scaling	n_estimators: [300,600,900]; max_depth: [None,10,20]; min_samples_leaf: [5,10,15]; max_features: ['sqrt',0.75,1.0].	max_depth=None; max_features='sqrt'; min_samples_leaf=10; n_estimators=600	1000
**Support Vector Regression (SVR)**	StandardScaler	kernel: ['rbf','linear']; C: [10,25,50,100]; epsilon: [0.05,0.1,0.2]; gamma (RBF): ['scale','auto',0.01,0.1].	C=50; epsilon=0.1; gamma='scale'; kernel='rbf'	Not applicable
**Multilayer Perceptron (MLP)**	StandardScaler	hidden_layer_sizes: [(256,256),(512,256),(512,512)]; activation: ['relu','tanh']; alpha: [1e-5,1e-4,1e-3]; learning_rate: ['constant','adaptive']; learning_rate_init: [0.0005,0.001,0.005]; solver: ['adam']; max_iter=1000.	activation='relu'; alpha=0.0001; hidden_layer_sizes=(512,512); learning_rate='constant'; learning_rate_init=0.001; solver='adam'	100
**Gradient Boosting**	No scaling	n_estimators: [100,200,300]; learning_rate: [0.025,0.05,0.1]; max_depth: [2,3,4]; subsample: [0.8,1.0]; max_features: [None,'sqrt']; min_samples_split: [2,4]; min_samples_leaf: [2,4,6]; loss: ['squared_error'].	learning_rate=0.05; max_depth=3; max_features=None; min_samples_leaf=4; min_samples_split=2; n_estimators=100; subsample=0.8	0
**XGBoost**	No scaling	n_estimators: [300,500,700]; max_depth: [3,5,7]; learning_rate: [0.025,0.05,0.1]; colsample_bytree: [0.8,1.0]; gamma: [0,1]; min_child_weight: [1,3,5]; reg_alpha: [0,0.1]; reg_lambda: [1,5]. Fixed: objective='reg:squarederror'; tree_method='hist'.	colsample_bytree=0.8; gamma=1; learning_rate=0.05; max_depth=5; min_child_weight=3; n_estimators=500; reg_alpha=0; reg_lambda=1	0

Note: The listed search spaces represent the parameter grids used for model tuning, and the selected values correspond to the optimal hyperparameters used for the final models. The internal holdout set remained separate from all tuning procedures.
